# Dynamic micropatterning uncovers spatial dynamics of B cell immune synapse formation

**DOI:** 10.1242/jcs.264876

**Published:** 2026-10-22

**Authors:** Blanca Tejeda-González, Sara Hernández-Pérez, Elmeri Kiviluoto, Johanna Ivaska, Pieta K. Mattila

**Affiliations:** ^1^ Institute of Biomedicine and MediCity Research Laboratories, University of Turku, 20014 Turku, Finland; ^2^ InFLAMES Research Flagship, University of Turku, 20014 Turku, Finland; ^3^ Turku Bioscience Centre, University of Turku and Åbo Akademi University, 20520 Turku, Finland; ^4^ Department of Life Technologies, University of Turku, 20014 Turku, Finland

**Keywords:** Immunological synapse, B cells, BCR signaling, Dynamic micropatterns, Microscopy

## Abstract

In the process of mounting humoral immune responses, B cells typically engage antigens on the surface of antigen-presenting cells at a site termed the immunological synapse (IS). To study this important cell interaction structure, we developed a dynamic micropatterning technique that models IS formation with precise spatial and temporal control. This method enables imaging of B cells before and after B cell antigen receptor (BCR) engagement, in fixed and live samples. We analyzed BCR-proximal signaling in A20 D1.3 mouse B cells and found distinct spatial distribution preferences for different signaling proteins. Also, the size and shape of the initial non-activatory adhesion modulated the signaling outcome. We visualized the formation of the IS in living cells using enhanced-resolution microscopy in three dimensions. We identified different cell behaviors during this process, including the repurposing of pre-existing actin-based protrusions as ready-made building blocks for the IS. Overall, dynamic micropatterning provides powerful insights into the rapid, early steps of IS formation with remarkable spatial and temporal resolution.

## Teaser

**Summary:** An adaptation of the dynamic micropattern method for studying early B cell activation reveals, in detail, how the immune synapse is formed and demonstrates segregation of different cellular signals in this process.

## INTRODUCTION

B lymphocytes (B cells), as a key component of the adaptive immune system, are responsible for mounting humoral immune responses against pathogens. Recognition of specific antigens via the B cell antigen receptor (BCR), along with additional co-stimulatory signals, initiates B cell activation and differentiation into antibody-producing plasma cells. It is increasingly recognized that, while soluble and particulate antigens can activate B cells, *in vivo* they typically encounter antigens that are displayed on the surface of antigen-presenting cells (APCs). This interaction leads to the formation of an intricate cell–cell contact known as the immunological synapse (IS) (Harwood and Batista, 2010; Huang et al., 2005; Kuokkanen et al., 2015; Tolar, 2017; Wykes et al., 1998), which shares strong similarity with the synapses originally described in T cell activation (Monks et al., 1998; Grakoui et al., 1999). The IS displays a bull's-eye-like structure with a central supramolecular activation cluster (cSMAC) where BCR-antigen complexes gather, and a peripheral SMAC (pSMAC), which consists of a radial, actin-rich protrusion enriched in adhesion molecules. The formation of the IS is initiated by the cell spreading over the antigen-presenting surface, enabling engagement with higher amounts of antigen, which is then extracted and endocytosed for antigen processing and presentation (Depoil et al., 2008; Fleire et al., 2006; Ketchum et al., 2014; McShane and Malinova, 2022; Spillane and Tolar, 2017). To study the formation and organization of this specialized structure, the IS can be modeled on various antigen-bearing surfaces *in vitro.* While using a model APC, such as Cos-7 cells expressing specific antigens on their surface (Fleire et al., 2006; Wang et al., 2018), provides the closest equivalent to *in vivo* conditions, particularly in the context of germinal center responses (Young and Brink, 2021), they present technical challenges for detailed analysis. As a result, many studies employ simplified setups with antigen-coated surfaces to recapitulate various aspects of the IS. Plastic or glass surfaces are most commonly used; however, lipid bilayers and polyacrylamide gels can better mimic important physical properties, such as surface fluidity and varying stiffness, respectively (Aceitón et al., 2025; McArthur et al., 2025). These different techniques that have been developed in the field have their specific advantages: supported lipid bilayers allow for the study of the clustering and movements of different membrane proteins, whereas gel systems enable the study of mechanical factors in B cell activation (Zeng et al., 2025). Despite the importance of the IS for lymphocyte activation, our understanding of the underlying mechanisms and regulation of the IS remains limited. In particular, early dynamics of this process, from the first cell–cell contact to spreading, are rapid and complex, with the initial contact and receptor engagement happening within seconds and full spreading completed within 10-20 min. As a result, studying these dynamic processes in detail has been challenging using traditional models.

In the past, micropatterning of substrates has been used to standardize the otherwise heterogeneous shape and size of living cells, enabling more systematic and accurate analysis of biological processes, including cell geometry, membrane tension and cytoskeletal dynamics (Huda et al., 2012; Théry et al., 2006). In immunology, micropatterns have also been used to study different aspects of T cell activation, including the effects of different co-stimulatory molecules and T cell receptor signaling (Motsch et al., 2019; Sadoun et al., 2021; Shen et al., 2008). Conventional, static micropatterns are inherently limited in their ability to model highly dynamic systems. A technique that allows precise temporal control of B cell activation is based on photoactivatable caged antigen (Wang et al., 2016). This method also allows the use of high-resolution imaging, but it requires specific equipment capable of inducing the photoactivation. To address these limitations, we have recently developed dynamic micropatterning, which utilizes biotin–streptavidin chemistry to functionalize, on demand, the non-adhesive regions surrounding the micropatterns. This method allows precise temporal control over the triggering of cellular processes of interest, while maintaining compatibility with various modalities of high-resolution microscopy for detailed spatiotemporal analysis (Isomursu et al., 2024).

In this work, we demonstrate the use of dynamic micropatterning to study IS formation using multiple ligands and varied pattern geometries. With this novel approach, we observed a surprisingly distinct localization of activated signaling proteins in the B cell synapse: phosphorylated CD79A (pCD79A) was enriched at the cell periphery, where new BCRs ligate to the antigen, while phosphorylated PLCγ2 (pPLCγ2) was concentrated in the non-activatory area in the IS center. Notably, the use of the dynamic micropatterns enabled high-resolution visualization of the earliest steps of IS formation using live imaging. This revealed an unexpected feature of B cell spreading mediated by pre-existing apical, actin-based veils. Altogether, our work demonstrates how dynamic micropatterns unlock new possibilities for studying early immune cell activation with both spatial precision and temporal resolution.

## RESULTS

### Dynamic micropatterns enable controlled modeling of the formation of the IS

Dynamic micropatterns were prepared by adapting a protocol from Isomursu et al. (2024) to model B cell IS formation (Fig. 1A). Briefly, microscopy coverslips were coated with hydrophobic PLL-PEG-biotin, and the desired shapes were printed onto the surface by UV irradiation through a photomask, rendering the resulting micropatterned areas hydrophilic again. The micropatterns were then coated with a mixture of fibronectin and fluorescently labeled fibrinogen to promote cell adhesion. After seeding the cells onto the micropattern, a second streptavidin (Strep)-conjugated substrate can be added to functionalize the surrounding area and activate the cells. For B cells, we used Fab fragments of an anti-IgM antibody (αIgM Fab) acting as a surface-bound surrogate antigen. We and others have previously shown that αIgM Fab fragments do not activate the BCR in soluble form (Isomursu et al., 2024; Volkmann et al., 2016).

**Fig. 1.**
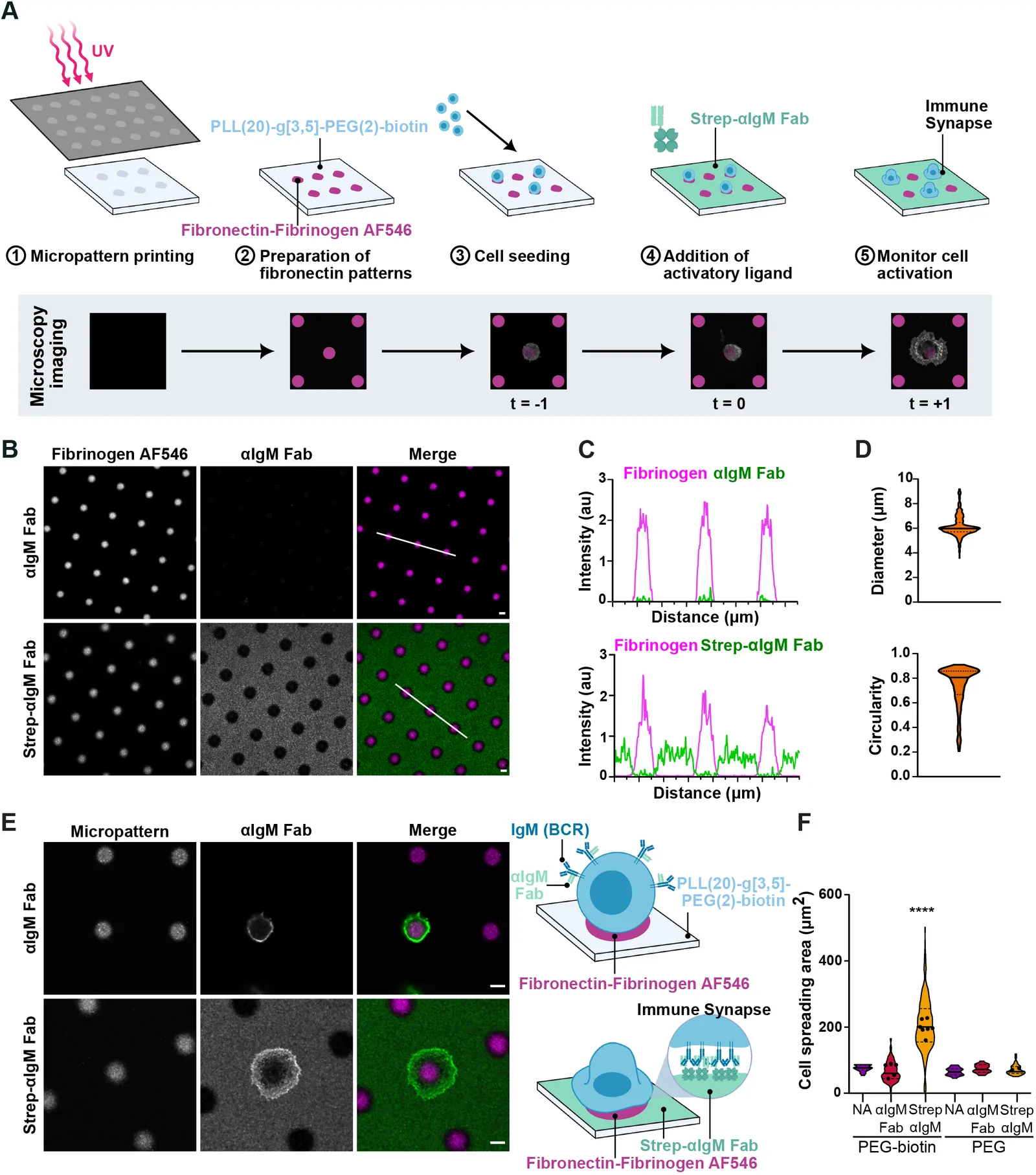
**Adapting dynamic micropatterns to model the B cell IS.** (A) Schematic presentation of the production of coverslips for dynamic micropatterning (top) and representative confocal images of the B cell activation in each step of the process (bottom). Created in BioRender by Mattila, P., 2026. https://BioRender.com/ega5fxb. This figure was sublicensed under CC-BY 4.0 terms. (B) Confocal representative images of 5 μm circular micropatterned coverslips with fibronectin and fibrinogen AF546 (magenta) coating and the binding of streptavidin-conjugated αIgM Fab AF488. Scale bars: 5 μm. (C) Intensity of the signal from fibrinogen and αIgM Fab along the lines marked in merged images of B (au, arbitrary unit). (D) Violin plot graph with mean (central line) and quartiles (dotted lines) of the diameter and circularity of the patterns as in B (*N*=3 experiments, *n*=800 patterns in total). (E) Representative confocal images of A20 D1.3 B cells seeded in 5 μm circular micropatterns (magenta) for 20 min and in the presence of unconjugated αIgM Fab (green) (top) or streptavidin-conjugated αIgM Fab (green) (bottom) for 15 min with a schematic of both conditions (right). Scale bars: 5 μm. (F) The quantification of the data shown in E. B cell spreading area measured by F-actin stained with phalloidin-670 during activation on PLL-PEG-coated or PLL-PEG-biotin-coated micropatterned coverslips. NA, non-activated. Violin plot graph with mean (central line), quartiles (dotted lines) and experiment means (dots) is shown. *N*=3-12 experiments. ****P<0.0001 (one-way ANOVA with Tukey's post-hoc test).

First, we verified the specificity and precision of substrate binding. As expected, fluorescently labeled fibrinogen was detected exclusively on the micropatterns generated by UV irradiation through the photomask (Fig. 1B,C). Correspondingly, when Strep-αIgM Fab fragments were added, they bound specifically to the PLL-PEG-biotin-coated areas surrounding the fibrinogen patterns. To examine the consistency and accuracy of pattern production, we measured the diameter and circularity of the micropatterns produced through a photomask with circular dot shapes of 5 μm in diameter (Fig. 1D). The resulting patterns were largely homogeneous, with an average diameter of approximately 6 μm, indicating that the irradiated area is slightly larger than the permissive hole in the mask, a typical consequence of the thin space left between the coverslip and the photomask upon irradiation.

We went on to test the performance of dynamic micropatterns to analyze the IS formation using A20 D1.3 cells, an IgM^+^ murine B cell line. A20 B cells are often used as a model cell line in IS studies (Burbage et al., 2017; Wang et al., 2022) and show a similar morphology to the IS of primary B cells and other cell lines (Liu et al., 2015; Roper et al., 2019). The cells were first allowed to adhere for 20 min to the circular micropatterns coated with fibronectin and fluorescently labeled fibrinogen. Fibronectin is a commonly used as a non-activating substrate for B cells (Rey-Barroso et al., 2018; Stupack et al., 1991; Treanor et al., 2010), which we mixed with fluorescent fibrinogen moieties for visualization. We tested the effects of this substrate on cell activation by immunoblotting and detected a slightly elevated phosphotyrosine (pTyr) signal compared to fibronectin alone, which was still very modest compared to the robust signaling detected in cells activated by BCR (Supplementary material, Fig. S1A,B). As expected, the unconjugated αIgM Fab fragments, added to the samples, bound to the BCRs on the cell membrane but did not adhere to either the patterned or unpatterned surface (Fig. 1E, top panels). Because of the lack of the BCR cross-linking and binding to the substrate, these unconjugated Fab fragments are unable to trigger BCR activation in solution (Fig. 1E,F). In contrast, Strep-conjugated αIgM Fab fragments robustly bound to the PLL-PEG-biotin, inducing activation as evidenced by cell spreading and IS formation (Fig. 1E, bottom panels, and Fig. 1F). These experiments demonstrate that only the combination of a permissive PLL-PEG-biotin surface and a surface-bound activatory ligand (Strep-αIgM Fab) can trigger IS formation (Fig. 1F), highlighting the specificity and suitability of this system for modeling IS formation under controlled conditions.

### Downstream BCR signaling proteins exhibit spatially distinct activation profiles in ligand-bound and unbound regions

Dynamic micropatterns offer a unique platform for directly comparing surface regions with or without BCR ligands, enabling us to investigate spatial signal segregation downstream of BCR engagement. To do this, B cells were seeded on fibronectin-coated micropatterns and activated with Strep-αIgM as described before for 0, 5, 15 or 30 min. Cells were fixed and stained with phospho-specific antibodies against pCD79A, phosphorylated Akt kinases (pAkt), pPLCγ2 and phosphorylated Btk (pBtk), as well as phalloidin to monitor cell spreading (Fig. 2A-C; Fig. S2A-C). The cells robustly spread on the Strep-αIgM ligand utilizing F-actin-based protrusions – smooth lamellipodia or spiky filopodia. BCR signaling is initiated by phosphorylation of CD79A, an integral part of the BCR complex bearing the immunoreceptor tyrosine-based activation motif (ITAM) and the site of the first detectable phosphorylation in response to antigen engagement. As expected, pCD79A was enriched at the peripheral edge of the spreading cell. Among the other signaling proteins analyzed, only pBtk showed a similar enrichment pattern, colocalizing with pCD79A at the cell periphery. In contrast, pPLCγ2 consistently accumulated in the central, non-activatory areas. pAkt transitioned from initial enrichment at the cell edge into a sparse, punctate distribution over the whole synaptic plane.

**Fig. 2.**
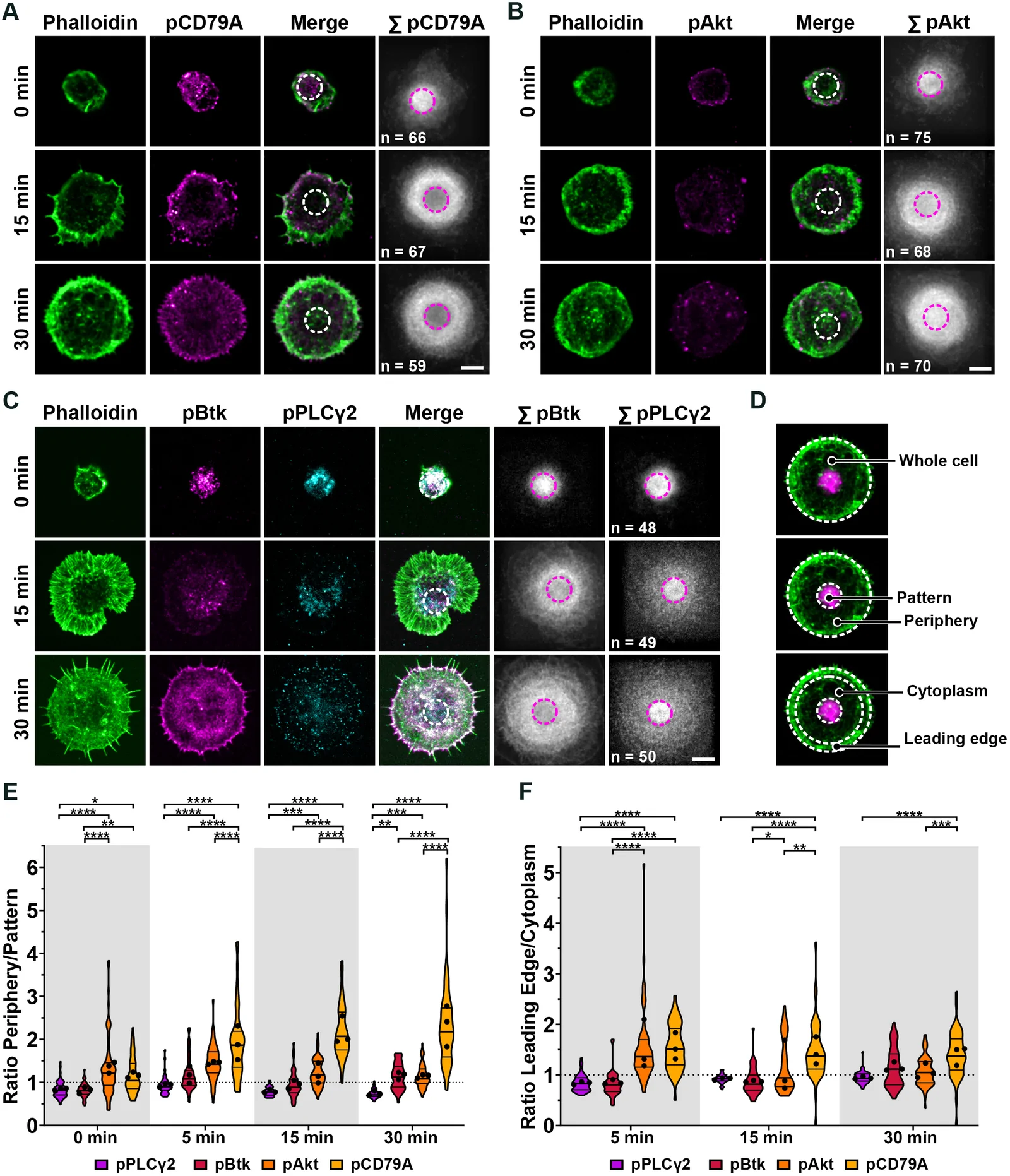
**Signaling proteins downstream of the BCR show differential localization in the IS.** (A-C) A20 D1.3 B cells were allowed to form ISs by first seeding them on fibronectin/fibrinogen-coated circular 5 μm (dashed white or magenta line) dynamic micropatterns for 15-20 min and then activated by the addition of Strep-αIgM Fab for 0, 15 or 30 min. Samples were fixed and stained for immunofluorescence analysis to visualize F-actin (phalloidin, green) and pCD79A (magenta; A), pAkt (magenta; B), pBtk (magenta; C) and pPLCγ2 (cyan; C). Representative images of single confocal microscopy slices are shown as well as summed images of phospho-proteins (white) of all cells per condition. 30 min summed images are also shown in Fig. S3. Scale bars: 5 μm. (D) Representation of the different ROIs analyzed from the data in A-C (F-actin, green; fibronectin, magenta): whole cell, pattern, periphery, cytoplasm, and leading edge. (E,F) The ratio of periphery RFI/pattern RFI (E) and the ratio of leading edge RFI/cytoplasm RFI (F) for all time points and phospho-proteins from the data shown in A-C. Violin plot graphs with mean (central line), quartiles (top and bottom lines) and experiment means (dots) are shown. *N*=3 experiments, *n*=48-75 cells. *P<0.05, **P<0.01, ***P<0.001, ****P<0.0001 (one-way ANOVA with Tukey's post-hoc test). RFI, relative fluorescence intensity.

To better visualize the distribution preferences of the phospho-proteins, we performed signal averaging across multiple cells using the fibronectin-coated micropattern as a reference point for alignment (Fig. 2A-C, right-most panels; Figs S2A-C, S3). These summed projections clearly demonstrated pPLCγ2 accumulation in the central fibronectin region, whereas pCD79A was strongly excluded from it. The peripheral enrichment of pCD79A and pBtk at the leading edge was not preserved in the summed projections, due to the variability in the orientation, size and morphology of cell spreading. pAkt showed initial enrichment to the site of BCR signaling but, by 30 min, the signal became uniform across the entire contact area, regardless of BCR ligand availability. While pBtk also showed exclusion from the fibronectin pattern, this effect was less pronounced than for pCD79A.

To analyze quantitatively the spatial distribution of signaling, we defined the following regions of interest (ROIs): whole cell; non-activatory pattern area; cell periphery; cytoplasm; and the leading edge (Fig. 2D). The analysis strongly supported the observations from both individual cells and signal-summed projections, supporting the differential localization of each phospho-protein during activation. Among the analyzed proteins, pCD79A showed the most distinct enrichment at the activatory area and leading edge. In contrast, pPLCγ2 was preferentially localized to the non-activatory patterned area, and pAkt and pBtk displayed intermediate behaviors (Fig. 2E,F).

To examine the possible effects of the non-activatory area on the dynamic micropatterns, we then compared the distribution and the intensity of the activated signaling proteins in the IS formed on the patterns and on coverslips that were only coated with PLL-PEG-biotin and Strep-labeled surrogate antigen. Notably, pAkt, pBtk and pCD79A all showed more central localization when seeded on uniform coating compared to cells on the micropatterns (Fig. S2D-F). pPLCγ2 distribution remained similarly enriched to the cell center both on patterns and the coated glass (Fig. S2G).

Together, these data suggest a substantial spatial uncoupling of BCR downstream signaling components from the initial site of receptor engagement.

### Mechanical strain conditions signaling responses to the BCR ligation

A benefit of micropatterns is that they can be generated in various sizes and shapes, allowing for the introduction of different mechanical stresses to the cell. With this in mind, we next used triangle-shaped patterns with 15 μm long edges, instead of the 5 μm circular micropatterns used above, to see whether a different mechanical cue with larger area and broken symmetry would affect the IS formation (Fig. S1C). The cells were left to adhere on the triangular patterns for 45 min longer than the circles, as they needed more time to adopt the shape and size of the triangles. Initiated from the triangles, the cells responded to the addition of Strep-αIgM with robust spreading, which, however, typically started on one of the three edges, causing more variation in cell shapes compared to the circular patterns (Fig. 3A-C; Figs S2A-C, S1D). The spreading area in the samples with triangles was larger than on the circles in the earliest time points, reflecting the larger starting point area, but equalized by 15 min (Fig. S1D,E).

**Fig. 3.**
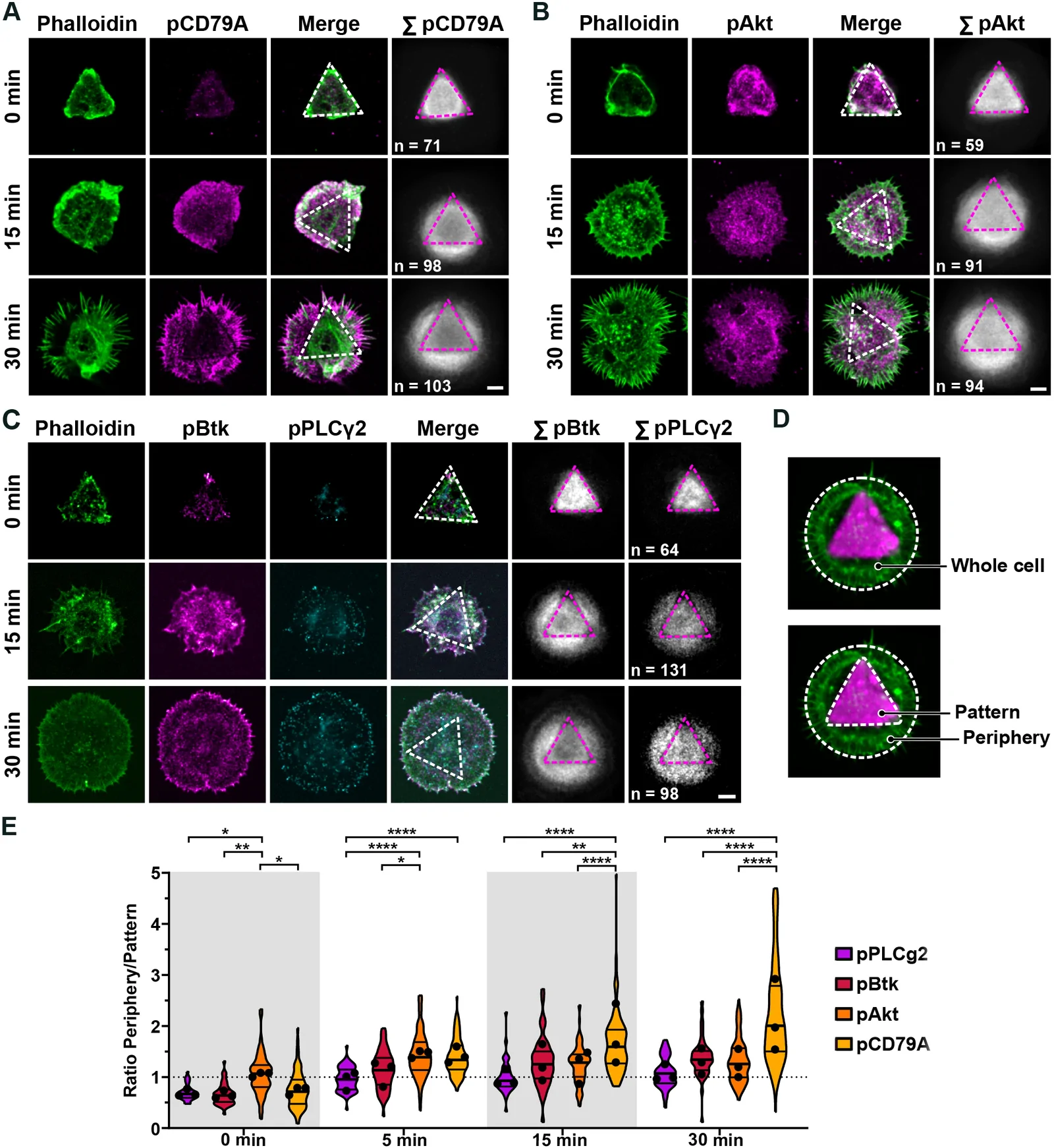
**BCR signaling proteins exhibit distinct responses to different micropatterns.** (A-C) A20 D1.3 B cells were allowed to form ISs by seeding them on fibronectin/fibrinogen-coated triangular 15 μm (dashed white or magenta line) dynamic micropatterns for 60 min and activated by the addition of Strep-αIgM Fab for 0, 15 or 30 min. Samples were fixed and stained for immunofluorescence analysis to visualize F-actin (phalloidin, green) and pCD79A (magenta; A), pAkt (magenta; B), pBtk (magenta; C) and pPLCγ2 (cyan; C). Representative images of single confocal microscopy slices are shown, along with the summed images of phospho-proteins (white) from all cells per condition and staining. 30 min summed images are also shown in Fig. S3. Scale bars: 5 μm. (D) Representation of the different ROIs analyzed in confocal images (F-actin, green) activated in 15 μm triangle micropatterns (fibronectin, magenta): whole cell, pattern and periphery. (E) The ratio of periphery RFI/pattern RFI in all time points and phospho-proteins from the data in A-C. Violin plot graphs with mean (central line), quartiles (top and bottom lines), and experiment means (dots) are shown. *N*=3 experiments, *n*=59-131 cells. *P<0.05, **P<0.01, ****P<0.0001 (one-way ANOVA with Tukey's post-hoc test). RFI, relative fluorescence intensity.

Cells seeded on triangular micropatterns showed largely similar distribution tendencies of the signaling proteins than the cells on circular patterns. pPLCγ2 showed a more uniform distribution than in the cells on circular patterns, but still did not become enriched at the activatory area. pCD79A, pBtk and pAkt showed enrichment towards the activatory area, pCD79A with the strongest preference to localize to the activatory area, as was also seen with the circular patterns (Fig. 3; Fig. S2A-C for the 5 min timepoint).

In contrast to the circular patterns, which cells could fully cover without any visible strain, the triangles caused the cells to mildly stretch when adopting the shape of the non-activatory pattern. When comparing these two different datasets, we detected some differences in the distribution of phosphorylation in activatory versus non-activatory areas. We could observe that pCD79A was less prevalent in the cell periphery in triangular patterns compared to circular patterns, whereas the other phospho-proteins showed an increase in their peripheral localization. The biggest change was detected in pPLCγ2, which exhibited a clear enrichment in the non-activatory area in synapses initiated from circles, but a uniform distribution throughout different substrates in triangle-initiated synapses (Fig. 3E; Figs S3, S4A).

To investigate the possible downstream effects of the pattern size and shape, we analyzed NFκβ translocation to the nucleus as a readout of productive cell activation (Kaileh and Sen, 2012). Cells seeded on both types of dynamic micropatterns for equal amount of time and activated for 30 min showed enrichment of NFκβ in the nucleus upon activation. This translocation was, however, more pronounced in cells seeded on triangular micropatterns, indicating that the spatial/mechanical conditions of the synapse also have an effect on downstream signaling events (Fig. S4B,C). Importantly, in this experiment we matched the seeding time of the cells in these two patterns to control for the possible effects of the adhesion time.

When comparing the cells on circles and triangles, we also noticed differences in the basal intensities of pPLCγ2 and pBtk. To test the effect of the cell confinement on the pattern, we compared the cells seeded for 60 min on circular or triangular patterns, or fibronectin-coated glass coverslips without patterns. We found that the pBtk levels on fibronectin-coated glass fell in between the levels on triangular and circular patterns. By contrast, pPLCγ2 intensity was found to be similar in cells seeded on a triangular pattern or an unpatterned surface, and significantly higher in cells seeded on circular patterns (Fig. S5A-D), indicating that B cells sense the confinement and react by modulating their signaling cascades.

Prompted by the observed differences in the circular and triangular patterns, we next tested the contribution of the initial incubation time by comparing cells seeded only on circular patterns but for either 20 or 60 min prior to activation. These experiments showed no significant differences in phospho-protein distributions between the activatory or non-activatory areas (Fig. S5E), indicating that the differences in the seeding times are unlikely to explain the differences between the two pattern conditions.

Finally, we went on to elucidate the contribution of the pattern size. For this, we added circular patterns with 10 μm diameter, with an area closer to that of the 15 μm triangular micropatterns (areas of all the micropatterns: 19.6 μm^2^ for 5 μm circles; 78.5 μm^2^ for 10 μm circles; and 97.5 μm^2^ for 15 μm triangles). Here, we again kept the seeding time constant (60 min in all three pattern conditions) to abolish the possible effects of time. Most significant differences in the distribution were seen in non-activated stages, where the larger pattern areas also occupied considerably more of the cells' volume. Upon activation, we saw significant differences in the distributions of pAkt and pPLCγ2 (only between the circles and triangles, not between the smaller and bigger circles), while pBtk and pCD79A did not show significant changes (Fig. S5F).

Although it was challenging to control all the variables in any single experiment, together these results indicated that the cells rather sense the triangular shape than the increase in size of the pattern or the seeding time, and that this can lead to altered phosphorylation and downstream events.

Together, our analysis showed that the distribution of phospho-proteins within the IS are influenced by the initial cell shape, dictated mostly by the selected micropattern geometry, with a smaller contribution of the pattern size. This suggests that factors such as mechanical constraints and cellular symmetry may contribute to the spatiotemporal regulation of the BCR activation cascade.

### Actin distribution in the synapse is affected by mechanical factors

Based on our hypothesis that the triangular pattern imposed mechanical constraints on cells, we went on to analyze the cytoskeletal organization of cells seeded on circles or triangles in greater depth. First, we studied the levels of phosphorylated myosin light chain, as a possible indicator of increased cellular tension, but did not detect any specific localization or alterations in cells on different patterns or upon activation, restricting its usability as a readout (data not shown).

Next, we turned into deeper analyses of the F-actin organization of cells seeded and activated on 5 μm circular or 15 μm triangular micropatterns (Fig. 4A). This analysis of the F-actin maximum intensity and skewness showed that the cells activated on triangular patterns form the brightest actin structures inside of the pattern instead of outside of it (Fig. 4B), as cells seeded in circular patterns do. All conditions had increased skewness, a tail of high values, in F-actin intensity (Fig. 4C) indicating that there are bright structures among an otherwise mostly uniform intensity profile. These high values were, however, more pronounced in cells seeded in triangular micropatterns, which indicates that the difference between the exceptionally bright structures and the average actin structures are significantly higher in cells seeded on triangles compared to the cells seeded on circular micropatterns. The same difference was seen both on the patterns and periphery. Our analysis fitted with the visual analysis in which we observed higher F-actin intensity ‘asters’ in the cells activated on triangles. Often, these asters were seen to localize to the corners and edges of the triangles (as in Fig. 4A, 5 min). Many cells also showed prominent F-actin bundles along the edges of the triangles (as in Fig. 4A, 15 min).

**Fig. 4.**
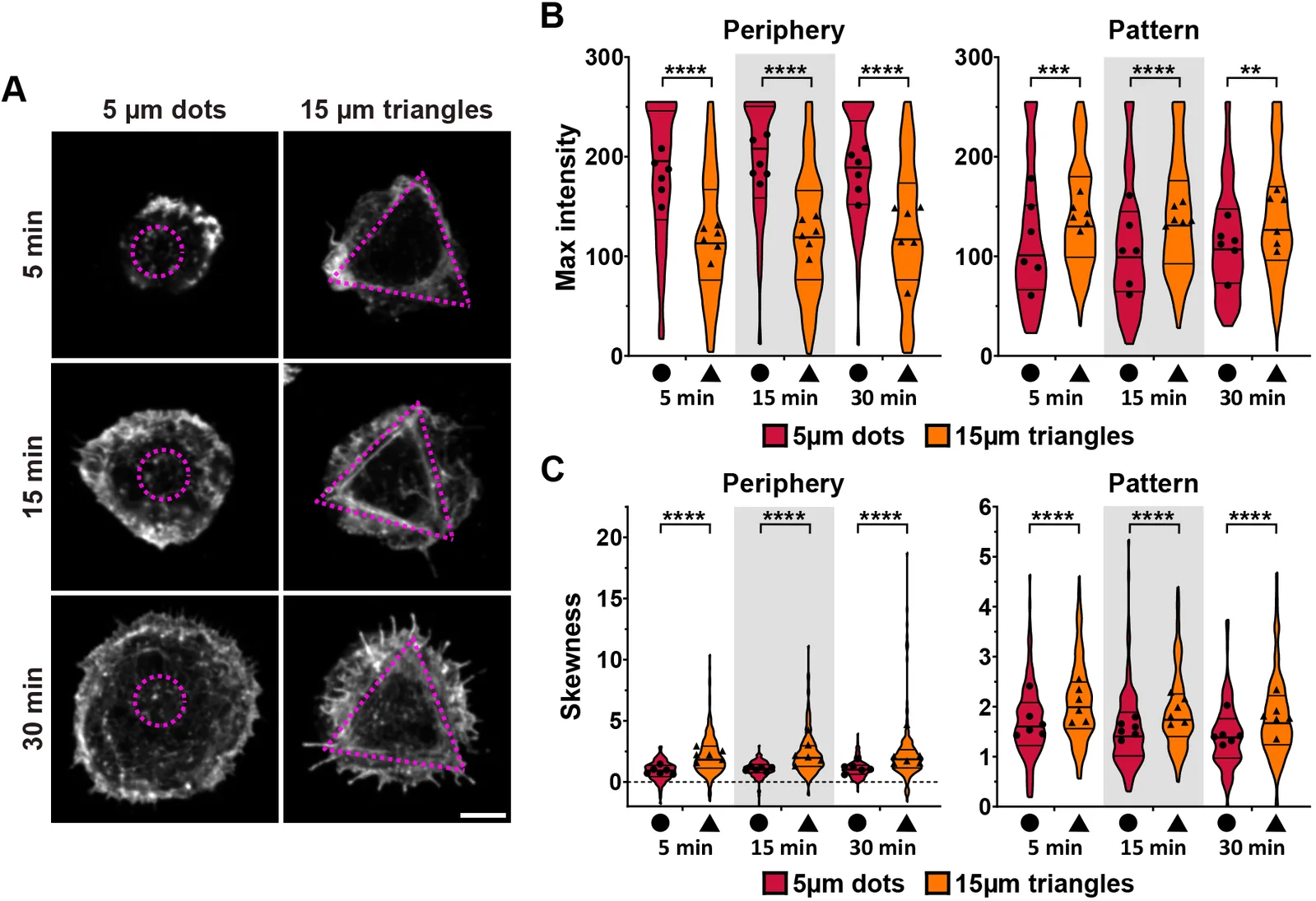
**Micropattern shape affects F-actin organization in the B cell IS.** The F-actin of the IS of A20 D1.3 B cells activated and fixed (Figs 2 and 3) was analyzed for characteristics of its distribution. (A) Representative images of single confocal microscopy slices stained for F-actin (phalloidin, white) and micropatterns (magenta outline). Scale bar: 5 μm. (B,C) Violin plots showing maximum intensity (B) and skewness (C) of actin values in the periphery and pattern areas of the IS. Violin plot graphs with mean (central line), quartiles (top and bottom lines) and experiment means (dots) are shown. *N*=6 experiments, *n*=134-200 cells. **P<0.01, ***P<0.001, ****P<0.0001 (one-way ANOVA with Šídák's post-hoc test).

It has been seen in other cell types that the constriction of cells to triangular areas leads to formation of F-actin structures that create traction forces in the same areas as we see in our cells, close to the periphery of the triangular micropattern (Amiri et al., 2023; von Erlach et al., 2018). Although we were not able to measure traction forces directly in our system, it could be postulated that the B cells settled on triangular patterns sense different mechanical forces than those on the circular patterns.

### Pre-existing protrusions guide early B cell synapse formation

Dynamic micropatterns offered a unique opportunity to examine the earliest stages of IS formation, starting from a resting, non-signaling state, with high spatiotemporal resolution. The delicate F-actin-rich protrusions are difficult to fully preserve in fixed samples. This led us to use live-cell imaging, utilizing the exogenous expression of the actin reporter LifeAct-Emerald, which enables visualization of the actin cytoskeleton in real-time.

Cells were seeded onto 5 μm fibronectin/fibrinogen circles and LifeAct-Emerald fluorescence was recorded before and for 15 min after activation with Strep-αIgM Fab. Using high-resolution three-dimensional (3D) confocal imaging, we tracked F-actin structures both at the apical side of the cell and the IS surface. We identified three distinct spreading phenotypes during IS formation: (1) 47% of the cells spread with smooth lamellipodia, (2) 24% of the cells used filopodia-driven spreading, and (3) 29% of the cells utilized lobular actin structures, generated from large, pre-existing actin ruffles from the apical side of the cells (Fig. 5A,D; Movie 1). Upon cell activation, these F-actin-based ruffles were seen to be rapidly dragged to the activatory surface to facilitate the formation of the IS (Fig. 5B). To our knowledge, such a ruffle phenotype has not previously been reported during early B cell activation and IS formation, most likely because the highly dynamic, 3D nature of these structures makes them difficult to visualize with traditional microscopy setups.

**Fig. 5.**
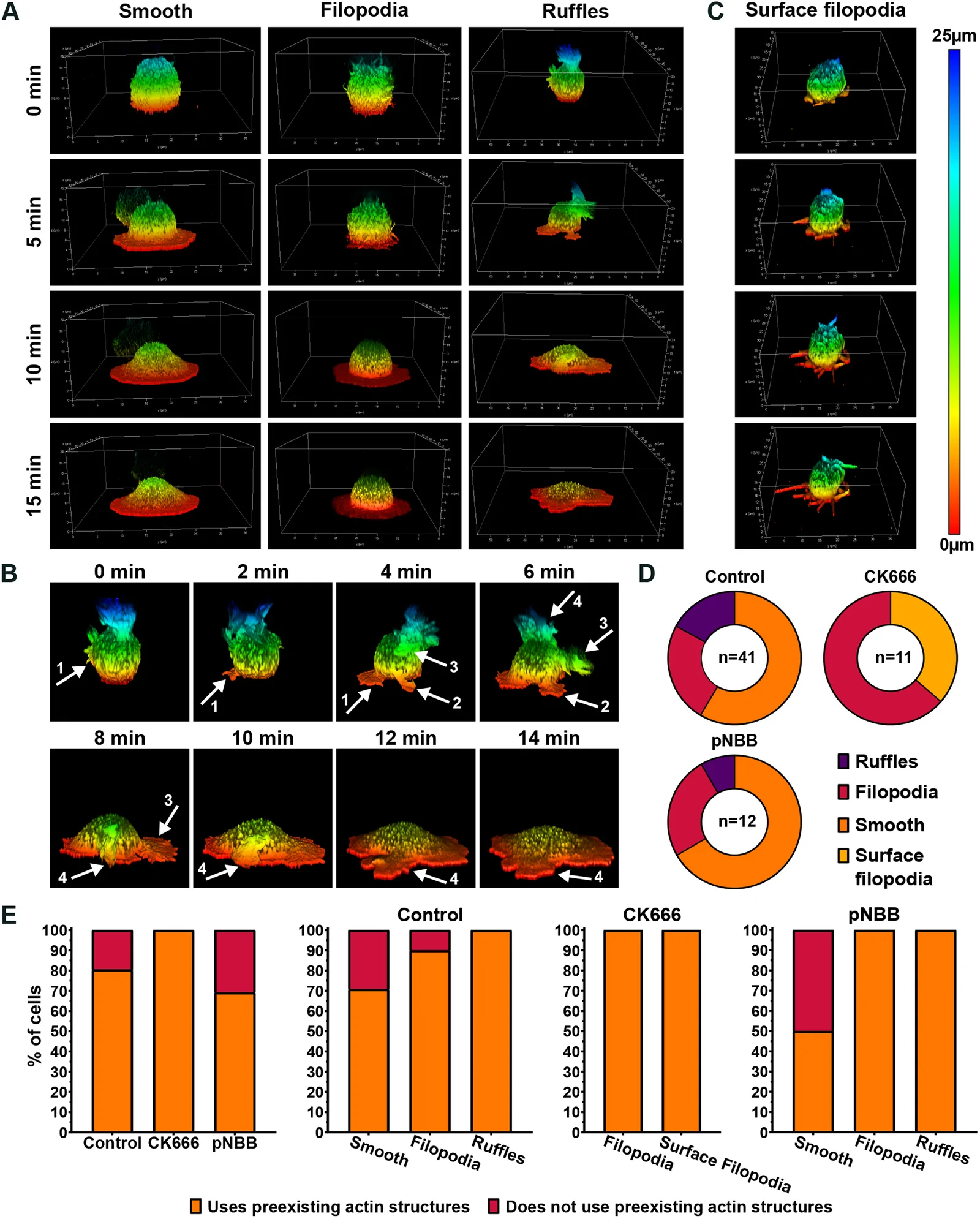
**B cells utilize pre-existing F-actin protrusions to facilitate cell spreading upon IS formation.** (A) A20 D1.3 B cells, transfected with LifeAct-Emerald, were seeded on fibronectin/fibrinogen-coated circular 5 μm dynamic micropatterns for 20 min and activated for 15 min by the addition of Strep-αIgM Fab with simultaneous imaging with a Leica Stellaris confocal microscope. Representative images of 3D confocal live imaging show different phenotypes: smooth spreading, filopodia-driven spreading, and actin ruffles-driven spreading. (B) Individual frames of a representative movie (Movie 1) of a cell with the actin ruffle phenotype shown in A (numbered arrows point to individual ruffles). (C,D) The cells were allowed to form ISs as in A, with the addition of the actin cytoskeleton inhibitors CK666 to inhibit Arp2/3 and para-nitro-blebbistatin (pNBB) to inhibit myosin II. (C) A representative cell, treated with CK666, showing the surface filopodia-driven spreading. (D) Pie charts of the number of cells showing each phenotype in all conditions. *n*=11-41 cells per condition. Data from *N*=3-11 experiments. (E) Stacked bar graphs of the percentage of cells that use their pre-existing actin structures for the spreading, shown per treatment (left graph) and per phenotype upon each cytoskeleton inhibitor treatment (control, CK666 and pNBB).

To understand the mechanisms behind these three cellular behaviors, we treated the cells with inhibitors of key cytoskeletal components: CK666 (an Arp2/3 inhibitor), which blocks branched actin formation required for lamellipodia, and para-nitro-blebbistatin (a myosin II inhibitor), which impairs actomyosin contractility. When Arp2/3 was inhibited, actin ruffles and smooth, lamellipodial spreading disappeared, leaving the cells to rely only on filopodia to spread and engage with the antigen (Fig. 5D; Fig. S6A). The inhibitor-treated cells showed two distinct sub-phenotypes. In 64% of the cells, filopodia similar to those seen in the non-treated cells were observed, which stemmed from the pre-existing filopodial, or microvillus-like, protrusions from the cell body and fused to the leading edge (Movie 3). However, 36% of cells showed particular filopodia, which we called surface filopodia, that were triggered on and extended only along the cell-surface contact (Fig. 5C; Movie 2). Conversely, when myosin II was inhibited, the most prevalent phenotype was the smooth spreading, and the number of ruffling cells decreased considerably (Fig. 5D; Movies 3-5, Fig. S6B). When analyzing the area of cell spreading upon the inhibitor treatments, we saw that myosin II inhibition did not affect the total spreading area, whereas cells treated with the Arp 2/3 inhibitor showed significantly decreased IS area (Fig. S6C,D).

Our live-cell microscopy data raised the question of whether the spreading phenotype of a specific cell could be determined by the actin structures already pre-existing in the cell before activation. To answer this, we analyzed the percentage of activated cells that used pre-existing structures similar to the spreading phenotype to form the IS. We found that over 80% of the untreated cells used the pre-existing actin structures, all of the CK666-treated cells used the pre-existing filopodia for their spreading, and 70% of the para-nitro-blebbistatin-treated cells used their pre-existing structures (Fig. 5E). When analyzing the data for each spreading phenotype, we saw that those cells that did not utilize their pre-existing actin structures spread by smooth lamellipodia, further suggesting lamellipodia as the *de novo* structure for spreading.

The changes in the prevalence of different phenotypes demonstrated the differential involvement of these cytoskeletal components in IS formation. Specifically, cell spreading utilizing pre-existing actin ruffles depended on both Arp2/3 activity, likely to create the ruffles, and on myosin-driven contractility, probably to pull the ruffles toward the IS. The use of pre-existing actin structures for spreading could be energetically cost-effective by minimizing the amount of cytoskeleton remodeling needed.

### Dynamic micropatterns support modeling of the IS across cell types and ligands

Finally, to explore the wider applicability of our approach, we expanded the use of dynamic micropatterns to model multiligand B cell stimulation. *In vivo*, B cells interact with other cells and the extracellular matrix using multiple different receptor–ligand interactions during their search for antigen and activation, as well as to find their niche in the lymph node (Bajénoff et al., 2006; Carrasco, 2023; Cinamon et al., 2008). For example, B cells interact with a range of extracellular matrix proteins, including fibronectin, collagen and laminin, via specific receptors on their surface (Diehl et al., 2025). Dynamic micropatterns could serve as a tool to model these combinatorial interactions by the addition of additional ligands to the patterned surface.

When B cells interact with an APC to form an IS, in addition to BCR-antigen binding, the integrin LFA-1 on the B cells binds to ICAM-1 on the APC surface (Carrasco et al., 2004; Wang et al., 2022). ICAM-1 has also been reported to be necessary for the migration of naïve B cells, when they polarize and move along the surface in the search for antigen, but it also elicits a decrease in migration speed at high concentrations as the adhesion becomes too strong (Sáez de Guinoa et al., 2011).

We modeled this interaction by adding ICAM-1 as a second Strep-conjugated ligand into our dynamic micropatterns. Activation was triggered by adding Strep-αIgM or Strep-ICAM-1 alone, or both at ratios of 2:1, 1:1 and 1:2. Cell spreading was analyzed alongside pCD79A levels (Fig. 6A). Engagement with ICAM-1 alone did not induce B cell spreading. Compared to Strep-αIgM alone, the 1:2 ratio of Strep-αIgM to Strep-ICAM-1 resulted in increased cell spreading, although with reduced overall pCD79A levels (Fig. 6B,C). This is likely a result of reduced BCR ligand availability when most of the biotin molecules carry ICAM-1 instead of the surrogate antigen. Conversely, the peripheral localization of pCD79A was enhanced by lower amounts of Strep-ICAM-1 (Fig. 6D).

**Fig. 6.**
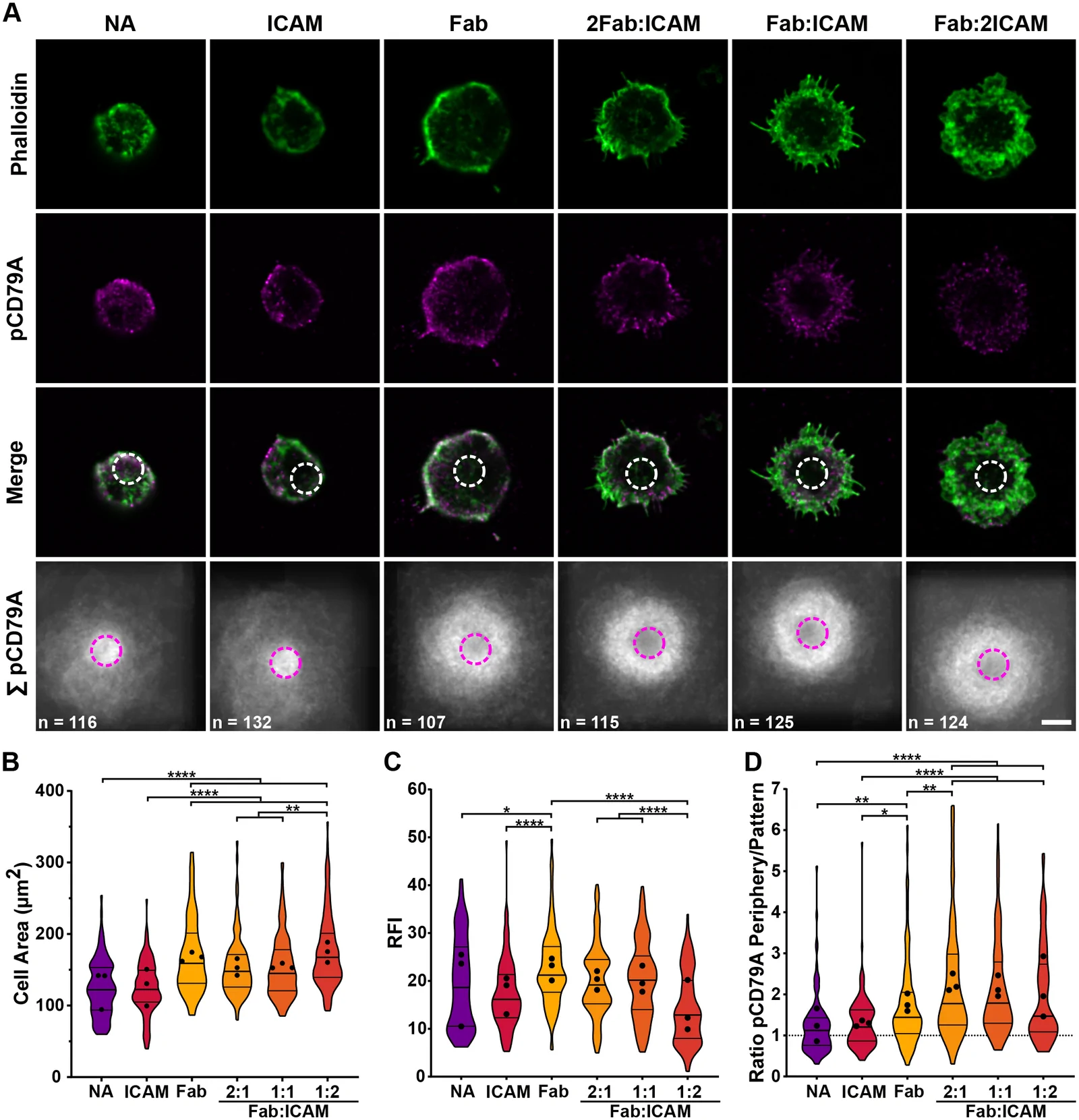
**Addition of multiple ligands in dynamic micropatterns supporting B cell IS formation, and adaptation of dynamic micropatterns for T cell IS formation.** (A) A20 D1.3 B cells were allowed to form ISs by seeding them on fibronectin/fibrinogen-coated circular 5 μm (dashed white or magenta lines) dynamic micropatterns and activating by the addition of different combinations of Strep-αIgM Fab and Strep-ICAM for 15 min. Samples were fixed and subjected for immunofluorescence analysis to visualize F-actin (green) and pCD79A (magenta). Representative images of single confocal microscopy slices are shown as well as summed images of phospho-proteins (white) of all cells per condition and staining. Scale bar: 5 μm. (B-D) Cell area (B), pCD79A mean intensity (C) and periphery RFI/pattern RFI ratio of pCD79A signal (D) from the data in A were analyzed. Violin plot graphs with mean (central line), quartiles (top and bottom lines), and experiment means (dots) are shown. *N*=3 experiments, *n*=107-132 cells. *P<0.05, **P<0.01, ****P<0.0001 (one-way ANOVA with Tukey's post-hoc test). NA, non-activated; RFI, relative fluorescence intensity.

The recognition and binding to ICAM-1 is mediated by downstream effects of early activation and cytoskeleton remodeling in the IS (Arana et al., 2008). ICAM-1 enhances adhesion to antigen-presenting surfaces and reduces the amount of antigen needed for activation, but it is not sufficient alone to mediate cell attachment to the surface (Carrasco et al., 2004). Our results align with the incapability of ICAM-1 alone to activate cells, and the enhancement of cell spreading at high doses corresponds with the increase of adhesion in the IS reported. With these results, we highlight the versatility of dynamic micropatterns for modeling IS formation across cell types and ligand combinations.

## DISCUSSION

Here, we adapt the recently developed dynamic micropatterning approach (Isomursu et al., 2024) to investigate IS formation with high spatial and temporal resolution. This method allows simultaneous analysis of IS regions with or without BCR engagement within a single cell. The technique enables high-resolution imaging of the earliest steps of lymphocyte activation on antigen-presenting surfaces. Our results reveal that activated signaling proteins exhibit distinct spatial distributions relative to activatory or non-activatory regions of the IS. Live-cell imaging further uncovered that, in addition to *de novo* actin polymerization, B cells repurpose pre-existing actin-rich protrusions as building material to facilitate spreading during IS formation.

When B cells form an IS on the surface of APCs, the recognized antigen is presented within a soft, laterally mobile membrane environment. Detailed study of IS on the native APC surface is technically challenging, particularly with microscopy-based approaches, and difficult to control. Various *in vitro* model systems have been developed to mimic IS formation, each with its own advantages and disadvantages, often resulting in a trade-off between physiological relevance and analytical power. For example, planar lipid bilayer systems have been developed to provide membrane fluidity (Dustin, 2009), while soft substrates simulate the mechanical properties of APCs (Aceitón et al., 2025; Zeng et al., 2015). By contrast, glass is very rigid and non-fluid, but highly amenable to high-resolution imaging and diverse functionalization strategies. Using dynamic micropatterns, we achieve a high level of spatial and temporal control over ligand presentation, and the ability to model the effects of different ligands in a straightforward manner, while remaining compatible with diverse imaging modalities, including super-resolution microscopy. Similar temporal control has previously been achieved using photoactivatable antigen (Wang et al., 2016), a method that requires specific equipment capable of inducing the photoactivation. Dynamic micropatterns also enable systematic exploration of co-stimulatory and co-inhibitory signals at the IS, as demonstrated by the addition of ICAM-1 in our experiments. The main limitation of the system is its relatively low throughput, as not every pattern attracts a cell, resulting in modest cell numbers per coverslip. In addition, different cell lines were found to present different compatibilities with the system. For example, the Raji human B cell line, as well as mouse primary B cells, showed poor adhesion to the micropatterns, potentially due to interference from the surrounding PLL-PEG matrix, warranting further optimization, such as non-activatory ligand on the original pattern that would better attract and adhere these cells than fibronectin. Our method is also limited in terms of the distribution of the antigen, which only reaches the periphery of the cell in the patterns used here. However, it could also be possible to use smaller patterns close to each other, allowing the cells to attach to several patterns simultaneously while leaving gaps for antigen entry in between. These kinds of smaller patterns have been used in studies of the T cell IS, with the activatory ligand in the patterned shape (Motsch et al., 2019; Sánchez et al., 2024; Shen et al., 2008).

When we analyzed the localization of phosphorylated signaling molecules (pPLCγ2, pBtk, pAkt and pCD79A) within the IS, we expected these signals to co-accumulate at the site of active BCR engagement. Instead, we observed distinct and non-overlapping patterns (Fig. 2A-C). pCD79A was enriched at the leading edge, consistent with this region being the site of new BCR-antigen engagement as the cell spreads to encounter new antigens. The decrease in signal toward the cell body likely reflects phosphatase-mediated negative feedback (Gold et al., 1991; Doody et al., 1995; Wienands et al, 1996). We also found pBtk mainly in activatory regions, highly enriched at the leading edge, aligning with its known role in actin remodeling via WASP (Sharma et al, 2009; Bhanja et al., 2022). Interestingly, pAkt showed only a slight preference for activatory areas, while pPLCγ2 strongly favored the non-activatory area of the IS when cells were seeded on the fibronectin/fibrinogen-coated circular patterns. Notably, on larger, triangular patterns, pPLCγ2 appeared more evenly distributed.

These results indicate that downstream BCR signaling rapidly diverges spatially within the IS, despite their reported connections, such as shared binding partners or complexes common to these proteins. For example, both PLCγ2 and Btk have been found to form a complex with BLNK downstream of CD79A activation (Fu et al., 1998; Hashimoto et al., 1999; Tanaka and Baba, 2020), yet they show opposite localization preferences regarding the BCR ligation status. Using fluid planar lipid bilayers as the antigen-presenting substrate, B cells as well as T cells have been shown to form ‘signaling microclusters’, where both receptor–antigen complexes and the proximal signaling molecules co-accumulate. However, the physiological plasma membrane of the APC presents variable diffusion for different proteins (Comrie et al., 2015), and microclusters become progressively less prominent as substrate fluidity decreases (Ketchum et al., 2014; McArthur et al., 2025).

Substrate mechanics introduce an additional layer of regulation. Mechanosensing of the activatory surface stiffness has emerged as an important factor in B cell activation, influencing processes such as IS formation (Aceitón et al., 2025), polarization (Pinon et al., 2022), activation threshold (Brooks et al., 2023) or antigen extraction (Spillane and Tolar, 2017). Here, we used small circular and larger triangular micropatterns to introduce different mechanical stresses to the cells at the onset of activation. F-actin structures in the IS showed differences between the two pattern shapes. The cells on triangular patterns formed prominent actin bundles and asters at the triangle vertices and edges, while cells on circular patterns showed more uniform F-actin structures that concentrated in the activatory area outside of the pattern (Fig. 4). Although not a direct measure of cellular tension, these results indicate that the cells sense different mechanical constraints on the patterns (Fig. 4). When comparing the signaling protein distributions between these two conditions, we observed shifts in the spatial organization of signaling proteins. It should be noted that not all these distributions behaved similarly across all the slightly different experimental setups (Figs S4A, S5F), suggesting some batch effects in the experiments. This indicates that there are other factors that contribute to these phospho-protein distribution changes that we do not completely understand or control. Nonetheless, our data indicate that various branches of BCR signaling respond differently to changes in the mechanical stress experienced by the cell. Importantly, we detected more-efficient NFκβ nuclear translocation in the cells seeded on triangular patterns compared to circles (Fig. S4B,C), suggesting that our model system can introduce mechanical signals that modulate the downstream cell activation. For instance, it is known that mechanical stretching can induce phosphorylation of Akt in a phosphoinositide 3-kinase (PI3K)-independent manner in other cell types (Kippenberger et al., 2005). Conversely, PLCγ2 and Btk have been linked to the ability of B cells to distinguish between soft and stiff substrates (Shaheen et al., 2017). Although the mechanisms of mechanosensing and its effects in B cells are still poorly understood, it could be speculated that the effects we observe are related, for example, to calcium signaling, as mechanosensing ion channels can influence calcium levels in B cells when in contact with glass surfaces (Kwak et al., 2023).

A key advantage of dynamic micropatterns is the precise synchronization of activation of cells pre-bound to non-activatory patterns, allowing high-resolution live imaging of the pre-selected cells already at the earliest stages of IS formation. If the cells are applied to the imaging chamber with a pre-existing antigen-coated surface, high-resolution imaging of the first steps of activation is challenged by the delay in finding the cells of interest in focus. Live imaging also enables visualization of delicate actin-based protrusions that are often lost during fixation. We effectively observed IS formation in three dimensions to examine the dynamics of the actin cytoskeleton during early activation. We identified three different phenotypes, characterized by the predominant actin structures formed during activation: smooth spreading, filopodia-driven spreading, and asymmetric spreading utilizing pre-existing apical actin ruffles. In the ruffle-based mode, cells appeared to recycle pre-existing F-actin and cell membrane as a whole to facilitate synaptic spreading, which could be interpreted as the utilization of pre-existing structures for more energy-efficient spreading upon activation. To our knowledge, this phenotype, which is dependent on Arp2/3 and myosin II contractility, has not been previously described in the formation of the B cell IS, but only during probing behaviors in resting B cells (Wang et al., 2016). Actin ruffles on B cells have been detected in B cells surveilling the environment when cells are exposed to soluble antigen (Cabrera-Reyes et al., 2025), and ruffle-like, actin-based probing structures during the exploration of surface-bound antigen (Sáez de Guinoa et al., 2011). The actin-based protrusions have been shown to gather antigen from the surface of the APCs and depend on the ARP2/3 complex to from (Bolger-Munro et al., 2019) but have not been imaged and analysed in 3D before. We believe that in our model the actin ruffles and filopodia formed by B cells before activation are probing structures, as seen in soluble activation and APC-mediated activation studies, but when antigen is added to the glass surface those structures are then recycled to form the lamellipodia-based IS seen frequently in studies that activate B cells on rigid structures. Our data underscore the ability of dynamic micropatterns to capture the earliest, mechanically and spatially complex phases of IS formation. Overall, these findings position dynamic micropatterns as a powerful platform for dissecting the spatial, mechanical and temporal mechanisms of IS formation with a level of precision that surpasses existing model systems.

## MATERIALS AND METHODS

Detailed information of antibodies, reagents and consumables used can be found in Tables S1 and S2.

### Cell lines

A20 D1.3 mouse lymphoma cells (Williams et al., 1994), which express a hen egg lysozyme-specific IgM BCR, were kind gifts from Professor Facundo Batista (the Ragon Institute of MGH, MIT and Harvard, USA). Cells were cultured in complete RPMI (cRPMI) [RPMI 1640 with 2.05 mM L-glutamine supplemented with 10% fetal calf serum (FCS) 5 μM 1468 β-mercaptoethanol, 4 mM L-glutamine, 10 mM HEPES and 100 U/ml penicillin/streptomycin)] and maintained at 37°C and 5% CO_2_.

### Western blotting

Forty-eight-well plates were coated with 200 μl of 10 μg/ml of goat anti-mouse IgM F(ab′)2 fragments (Jackson ImmunoResearch, 115-006-020), 10 μg/ml fibronectin (F4759, Sigma-Aldrich) or 10 μg/ml combination of fibronectin and fibrinogen AF546 (F13192, Thermo Fisher Scientific) for 2 h at 37°C. A20 D1.3 B cells were starved for 15 min in RPMI medium supplemented with 0.5% FCS at 3×10^6^ cells/ml; 500,000 cells were seeded per well and stimulated for 15 or 30 min at 37°C and 5% CO_2_. After stimulation, cells were instantly lysed with 5× SDS lysis buffer (to a final concentration of 62.5 mM Tris-HCl, pH ∼6.8, 2% SDS, 10% glycerol, 100 mM β-mercaptoethanol, and Bromophenol Blue) and sonicated for 5 min (high power, 30 s on/off cycle; Bioruptor Plus, Diagenode). Lysates were run on 10% polyacrylamide gels and transferred to PVDF membranes (Trans-Blot Turbo Transfer System, Bio-Rad). Membranes were blocked with 5% bovine serum albumin (BSA) in TBS (pH ∼7.4) for 1 h and incubated with anti-phosphotyrosine (1:1000; 05-321, Merck) in 5% BSA in TBST (TBS with 0.05% Tween-20) at 4°C, overnight. After washing, horseradish peroxidase (HRP)-conjugated antibodies (1:10,000) were incubated for 1 h at room temperature (RT) in 5% milk in TBST. After a final wash, membranes were visualized with Immobilon Western Chemiluminescent HRP Substrate (WBKLS0500, Millipore) in a ChemiDoc MP Imaging System (Bio-Rad). Blots were stripped with 25 mM glycine-HCl buffer, 2% SDS, pH ∼2.5, for 10 min at 37°C, blocked, and re-probed with anti-β-actin antibody (HRP) (1:20,000; AC-15, Abcam, ab49900). Washing steps were carried out with 10 ml of TBST for 5×5 min. For quantification, images were background subtracted, and the raw integrated densities of the lanes (pTyr) were normalized to corresponding total β-actin densities in ImageJ. Images of the full western blot membranes are shown in Fig. S7.

### Ligand conjugation

Anti-IgM Fab (Jackson ImmunoResearch, 115-007-020) and ICAM-1 (BioLegend, 553004) were conjugated to Strep using the Streptavidin Conjugation Kit Lightning-Link (Abcam, ab102921) following the manufacturer's instructions.

### Micropatterned coverslip production

Round coverslips (13 mm or 25 mm diameter, 0.16-0.19 mm thickness; Epredia) were prepared as previously described (Isomursu et al., 2024). Coverslips were first cleaned with 70% and 94% ethanol consecutively and then placed in a UV oven (UVO-cleaner 342-220, Jelight Company; low-pressure mercury vapor lamp, fused quartz, λ=185 and 254 nm, 3033 mW cm^−2^ at 254 nm with a distance of ¼′′) for 15 min, by which time all the ethanol had evaporated from the glass surface. Coverslips were coated in a wet chamber with 0.1 mg/ml PLL(20)-g[3.5]-PEG(2)/PEG(3.4)-biotin(50%) (SuSoS, Switzerland) in dH_2_O for 1 h at RT, washed twice in PBS and once in dH_2_O.

Coated coverslips were then patterned using quartz/chromium photomasks with areas for 5 μm circular micropatterns 10 μm circular micropatterns and 15 μm side equilateral triangular micropatterns (Delta Mask, Netherlands). Photomasks were cleaned with dH_2_O, followed by 70% and 94% ethanol, dried using airflow and then cleaned in the UV oven for 5 min. Coverslips were placed on the photomask, inverted on a drop of dH_2_O and exposed to UV light for 6 min. The patterned coverslips were stored at 4°C until use.

A maximum of 24 h before use, patterned coverslips were coated with 10 μg/ml fibronectin (Sigma-Aldrich, F4759) and fibrinogen-Alexa Fluor (AF) 546 (1:200; Thermo Fisher Scientific, F13192) in PBS for 1 h at RT, washed twice in PBS and stored in PBS at 4°C until use.

### Immunofluorescence sample preparation

Micropatterned 13 mm coverslips were prepared as described in the ‘Micropatterned coverslip production’ section and placed in 24-well plates with 300 μl of starvation RPMI (sRPMI) (RPMI 1640 with 2.05 mM L-glutamine supplemented with 2% FCS, 5 μM 1468 β-mercaptoethanol, 4 mM L-glutamine, 10 mM HEPES and 100 μ/ml penicillin/streptomycin). A20 D1.3 cells were centrifuged at 700 rpm (1045 ***g***) for 5 min and resuspended at 10^6^ cells/ml in sRPMI; 200,000 cells were seeded per coverslip for 15 min at 37°C and 5% CO_2_. After seeding, coverslips were washed twice with sRPMI to remove unbound cells. Cells seeded in 5 μm dot micropatterns were then activated using 0.5 μl of Strep-anti-IgM Fab (stock 1 mg/ml), 1 μl of Strep-ICAM (stock 0.5 mg/ml), or a mixture of both at different ratios (2:1, 1:1 and 1:2) per coverslip. The cells seeded in 10 μm circular and 15 μm triangle micropatterns were, after washing off the unbound cells, allowed to take the shape of the pattern for an additional 45 min at 37°C and 5% CO_2_, to reach total of 60 min seeding time, and then activated as above. In experiments for which it is indicated, cells in 5 μm circular micropatterns were seeded for the same period of time as cells in 10 μm circular and 15 μm triangular micropatterns. After 5, 15 or 30 min of activation cells were fixed using 4% paraformaldehyde (PFA) for 10 min at RT and washed twice in PBS.

For non-patterned control samples, 12-well slides (Epredia, X1XER302W) were prepared in the same manner as coverslips for micropatterning. Wells were cleaned with 70% and 94% ethanol consecutively and then placed in a UV oven (UVO-cleaner 342-220, Jelight Company; low-pressure mercury vapor lamp, fused quartz, λ=185 and 254 nm, 3033 mW cm^−2^ at 254 nm with a distance of ¼′′) for 15 min, by which time all the ethanol had evaporated from the glass surface. Coverslips were coated in a wet chamber with 0.1 mg/ml PLL(20)-g[3.5]-PEG(2)/PEG(3.4)-biotin(50%) (SuSoS, Switzerland) in dH_2_O for 1 h at RT, then washed twice in PBS and once in dH_2_O. Non-activatory control wells, with no PLL-PEG coating, were coated with 10 μg/ml fibronectin (Sigma-Aldrich, F4759) and fibrinogen-AF546 (1:200; Thermo Fisher Scientific, F13192) in PBS and PLL-PEG coated wells were coated with Strep-anti-IgM Fab (stock 1 mg/ml) at 1:1200 in PBS for 1 h at RT, washed twice in PBS and stored in PBS at 4°C until use. A20 D1.3 cells were centrifuged at 700 rpm (1045 ***g***) for 5 min and resuspended at 1.5×10^6^ cells/ml in sRPMI; 30,000 cells were seeded per well and activated for 5, 15 or 30 min at 37°C and 5% CO_2_. After activation, cells were fixed using 4% PFA for 10 min at RT and washed twice in PBS.

In a wet chamber, samples were permeabilized and blocked (5% donkey serum, 0.3% Triton X-100 in PBS) for 20 min at RT. After blocking, cells were incubated with primary antibodies (Table S1) and ActiStain-488 Phalloidin (Cytoskeleton, PHDG1-A), ActiStain-555 Phalloidin (Cytoskeleton, PHDH1A) or Phalloidin-iFluor-405 (Abcam, ab176752) at 1:200 in staining buffer (1% BSA, 0.3% Triton X-100 in PBS) at 4°C overnight and then washed in PBS to remove unbound antibodies. Cells were then incubated in donkey anti-rabbit IgG AF647 (Invitrogen, A31573) secondary antibody at 1:500 in PBS for 45 min at RT, washed in PBS and mounted using Fluoromount G with (Invitrogen, 00495952) or without (Southern Biotech, 0100-01) DAPI.

### Basal activation

For basal activation, 13 mm micropatterned coverslips were prepared as described in the ‘Micropatterned coverslip production’ section. Coverslips were coated with 10 μg/ml fibronectin (Sigma-Aldrich, F4759) and fibrinogen-AF546 (1:200; Thermo Fisher Scientific, F13192) in PBS for 1 h at RT for unpatterned control samples. A20 D1.3 B cells were seeded and washed as described in the ‘Immunofluorescence sample preparation’ section; cells for all conditions were left adhered on the patterns for 1 h at 37°C and 5% CO_2_. Cells were then fixed using 4% PFA for 10 min at RT and then washed twice in PBS. Samples were stained as described in the ‘Immunofluorescence sample preparation’ section.

### Cytoskeleton inhibitor treatment

Immunofluorescence samples were prepared as described in the ‘Immunofluorescence sample preparation’ section with the additional step of treatment of cells with CK666 100 μM 30 min (Sigma-Aldrich, 182515), para-nitro-Blebbistatin 25 μM 30 min (Cayman Chemical, 24171) or DMSO after cell seeding on the micropatterned coverslips.

### Pattern quality control and characterization

Micropatterned coverslips (5 μm dots, 13 mm diameter coverslips) were produced as described in the ‘Micropatterned coverslip production’ section, and anti-IgM Fab (Jackson ImmunoResearch, 115-007-020) or anti-IgM Fab conjugated with Strep was added. Cells were then washed twice in PBS and stained with donkey anti-goat IgG AF488 (Thermo Fisher Scientific, A-11055) at 1:500 in PBS for 1 h RT in a wet chamber.

For the control of B cell activation on different surfaces, A20 D1.3 cells were seeded as previously in the ‘Immunofluorescence sample preparation’ section on coverslips coated with PLL-PEG-biotin (SuSoS, Switzerland) or PLL-PEG and micropatterned as previously described. Cells were then activated with anti-IgM Fab (Jackson ImmunoResearch, 115-007-020) or Strep-conjugated anti-IgM Fab for 15 min at 37°C and 5% CO_2_. Cells were then fixed with 4% PFA for 10 min at RT, washed twice in PBS, blocked (5% donkey serum, 0.3% Triton X-100 in PBS) for 20 min RT, stained with donkey anti-goat IgG AF488 (Thermo Fisher Scientific, A-11055) at 1:500 or ActiStain-670 Phalloidin (Cytoskeleton, PHDN1-A) at 1:200 in PBS for 1 h RT in a wet chamber, washed twice with PBS and mounted using Fluoromount G (Southern Biotech, 0100-01).

### Live imaging

A20 D1.3 cells were transfected as previously described (Šuštar et al., 2018). Cells suspended in 2S transfection buffer (5 mM KCl, 15 mM MgCl_2_, 15 mM HEPES, 50 mM sodium succinate, 180 mM Na_2_HPO_4_/NaH_2_PO_4_, pH 7.2) were transfected with 1 μg of plasmid per 10^6^ cells and electroporated using an AMAXA electroporation machine (program X-005, Biosystem) in 0.2 cm gap electroporation cuvettes. LifeAct-Emerald plasmid was a kind gift from Guillaume Jaquemet (Åbo Akademi University, Turku, Finland).

Micropatterned 25 mm coverslips in cell chambers (Invitrogen, A7816) were seeded as described in the ‘Immunofluorescence sample preparation’ section with transfected cells 12 to 24 h after transfection. Just before imaging, unbound cells were washed with chamber buffer (0.5% FCS, 2 mM Mg^2+^, 0.5 mM Ca^2+^, 1 g/l D-glucose in PBS). Imaging was started using a Leica Stellaris 8 confocal microscope (3D imaging) and after 1 min activation was initiated as described in the ‘Immunofluorescence sample preparation’ section without stopping the imaging. Activation was recorded for 15 min. When used, cytoskeleton inhibitors were added for pretreatment before imaging (CK666: 100 μM, 30 min; para-nitro-blebbistatin: 25 μM, 30 min).

### Image acquisition

#### Spinning disk confocal microscopy, fixed samples

Images were acquired with a 3i CSU-W1 spinning disk equipped with 405, 488, 561 and 640 nm solid state lasers and 445/45, 525/30, 617/37 and 692/40 nm filters and a 63× Zeiss Plan-Apochromat objective. A Photometrics Prime BSI sCMOS (2048×2084 pixels, 1×1 binning) camera was used.

#### Leica Stellaris live imaging

Images were acquired with a Leica Stellaris 8 Falcon FLIM equipped with a white light laser and a 405 diode laser and a HC PL APO 63×/1.40 OIL CS2 objective. Power HyD S SP pos 2, Power HyD S SP Core Unit Pos 3, Power HyD X SP pos 4 and Power HyD R SP pos 5 detectors were used.

### Image analysis and processing

#### Spinning disk confocal microscopy data

IS plane images were analyzed using Fiji ImageJ. IS plane images were thresholded using the automatic threshold Huang 2 in the F-actin and the fibronectin-fibrinogen channels to create the necessary ROIs for the analysis of the mean fluorescence intensity (MFI) of the different phospho-proteins in the full cell, periphery, pattern, leading edge and cytoplasm regions as well as to calculate the ratios between regions. The leading edge ROI was created by isolating a 2 μm stripe at the border of the cell. Leading edge to cytoplasm analysis was not performed on triangle patterns as a large fraction of the cells did not form such well-organized lamellipodia as the cells spreading from the circles, inhibiting confident thresholding of the leading edge for automated analysis. F-actin analysis was performed following the same procedure but analyzing skewness and maximum intensity of the F-actin channel instead. In unpatterned cells, the center of the IS was determined using the F-actin channel, and a circle of the same area as a 5 μm dot micropattern was drawn, creating the center and periphery ROIs for these images.

Sum images were created by aligning all images per condition using Fast4Dreg alignment on the micropattern channel, applying drift correction to all channels, normalizing signal and then producing a sum image of the stack. The process for the creation of summed images has some limitations and can produce some artifacts when the alignment introduces an area without any signal, leading to enhanced background in the rest of the area (visible in Fig. 6, Fab:2ICAM). The hazy signal surrounding the pattern in the non-activated cells is caused by tilting of some of the cells on the PLL-PEG surrounding the patterns upon sample fixation.

For the analysis of NFκβ translocation, a single plane in the middle of the nucleus was selected, and F-actin and DAPI channels were thresholded using the automatic threshold Huang 2 to create the ROIs for the analysis of the ratio of MFI between nucleus and cytoplasm.

#### Leica Stellaris data

Live imaging was processed using Las X adaptive Lightning processing adjusted for water medium to enhance signal. Phenotypes were described and quantified manually by the observation of the most prominent actin features during activation and spreading; this scoring was performed by a researcher who was aware of the treatment groups and who was familiar with the experimental design and cell behavior. IS area was analyzed using Fiji ImageJ by isolating the bottom three to five *z*-slices of the stack and creating a sum *z*-projection of the planes. The resulting image was thresholded using the automatic threshold Huang 2 to create the IS ROI that was then measured at all time points.

### Statistical analysis and illustrations

Statistical significance was calculated using ordinary one-way ANOVAs or unpaired Student's *t*-test assuming normal distribution of the data. Statistical significances are denoted as follows: *P<0.05, **P<0.01, ***P<0.001, ****P<0.0001. Statistical analysis was carried out and graphs generated in GraphPad Prism 8, and illustrations were created using BioRender or Inkscape v1.2. Figure formatting was also performed using Inkscape v1.2.

### Use of artificial intelligence

Grammarly was used to improve the grammar. The authors subsequently reviewed and edited the content as necessary and take full responsibility for the publication's final content.

## Supplementary Material

10.1242/joces.264876_sup1 Supplementary information
