# Supplementary material for: Dynamic micropatterning uncovers spatial dynamics of B cell immune synapse formation
Source: J Cell Sci. 2026 Oct 22;139(21):jcs264876. doi: 10.1242/jcs.264876 (PMC13723395; doi:10.1242/jcs.264876)
Supplement: Supplementary file 1 [file joces-139-264876-s1.pdf]

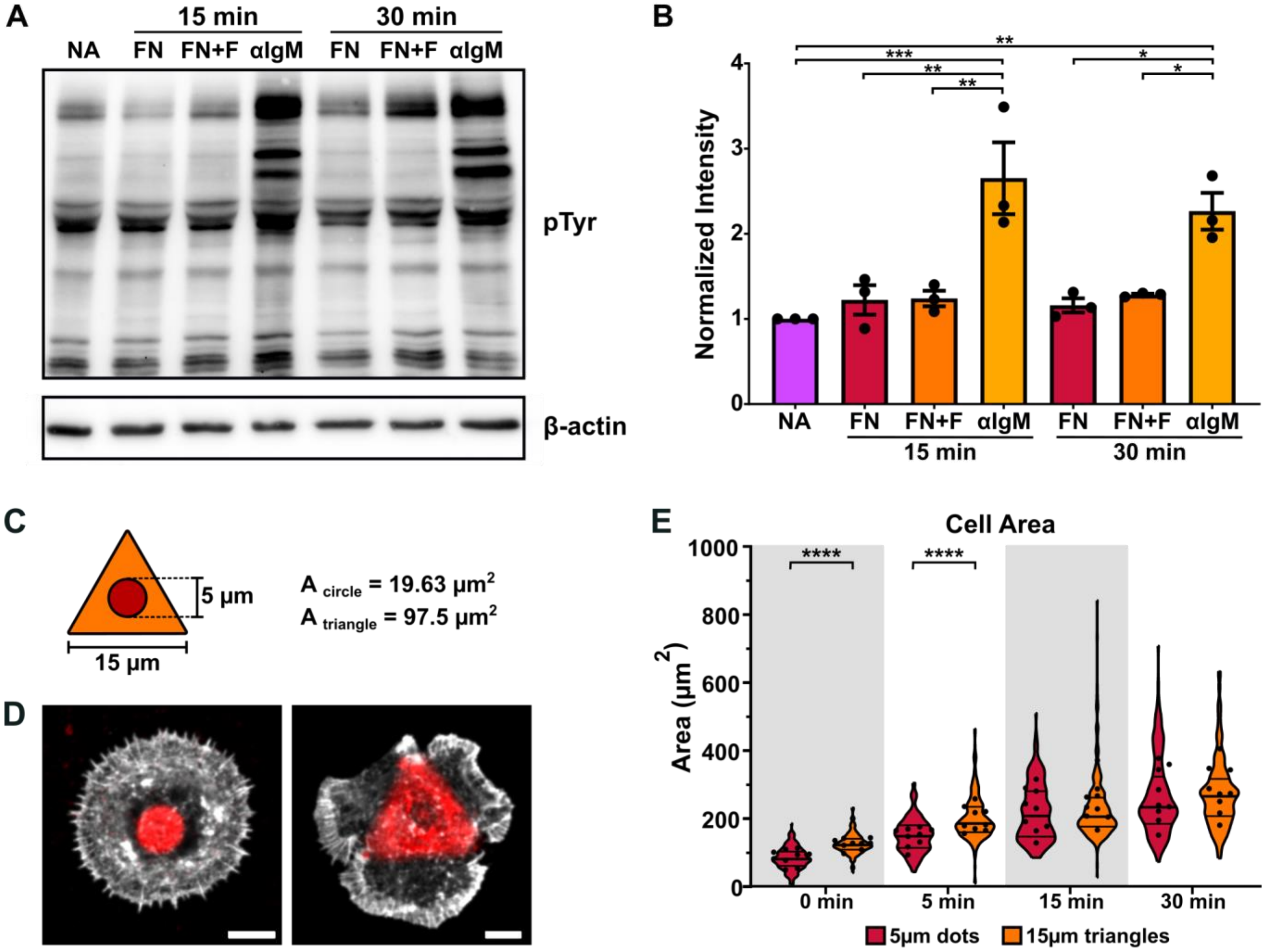

Fig. S1. B cells are only weakly activated by the substrate with combination of fibronectin and fibrinogen and IS formation in circular and triangular micropatterns. (A) A20 D1.3 B cells were seeded on glass coverslips left uncoated (NA) or coated with fibronectin (FN), fibronectin and fibrinogen AF546 (FN+F) as used for dynamic micropatterns, or αIgM Fab2 (αIgM). After 15 or 30 minutes, the samples were lysed and immunoblotted with HRP-anti phospho-tyrosine and anti-β-actin as loading control. (B) Bar graph of the quantification of normalized intensity with mean, SEM and individual experiment values (dots). Values are shown as change compared to non-activated cells after normalization of values using the β-actin loading control. One-way ANOVA (N=3 experiments). (C) Representative drawings of the sizes of 5 µm circular and 15 µm triangle micropatterns to scale. (D) Representative confocal images of A20D1.3 B cells let to activate for 30 min from 5 µm circular or 15 µm triangle micropatterns (fibronectin, red) fixed and stained for F-actin (white). Scale bar 5 µm. (E) Violin plot graphs with mean (central line), quartiles (top and bottom lines) and experiment means (dots) of cell area in the IS measured by F-actin staining. One-way ANOVA (N=9 experiments, n=135-205 cells) (*P < 0.05, **P < 0.01, ***P < 0.001, ****P < 0.0001).

Journal of Cell Science • Supplementary information

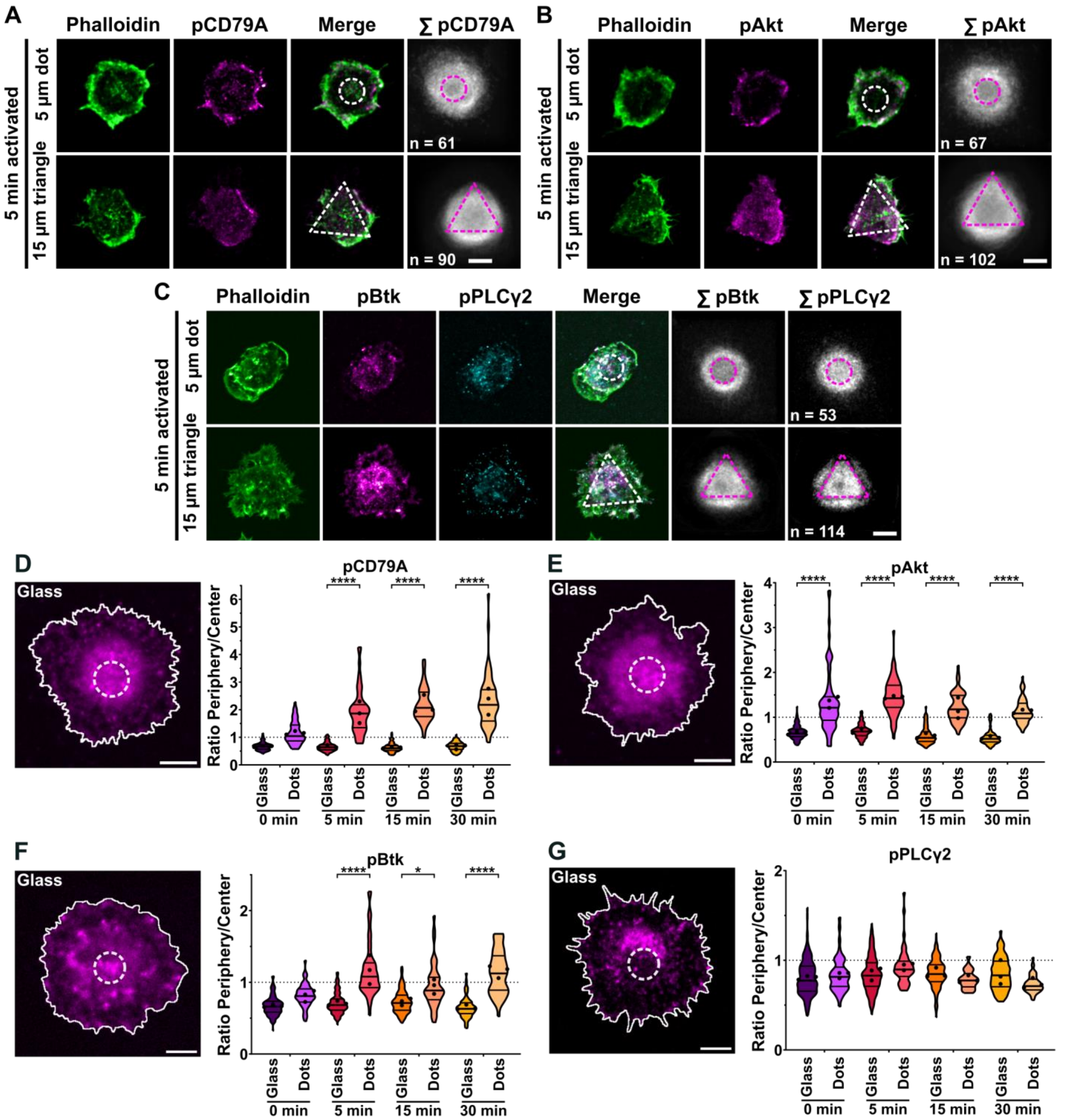

Fig. S2. Activation on unpatterned vs. patterned surfaces produces differences in signaling protein distribution. (A-C) Representative images corresponding to 5 minute activated cells in Figures 2 and 3. Scale bar 5 µm. (D-G) A20 D1.3 B cells were let to form immune synapses via seeding them on fibronectin/fibrinogen-coated circular 5 µm dynamic micropatterns 20 minutes and activated by the addition of Strep-αIgM Fab for 0, 5, 15 or 30 minutes (Figure 2) or seeded on PLL-PEG-biotin + Strep-αIgM Fab coated glass slides and activated for 0, 5, 15 or 30 minutes. Samples were fixed and stained for pCD79A (A), pAkt (B), pBtk (C) and pPLCγ2 (D). The center of the IS was calculated and a circular area the same size of the 5 µm circular micropatterns was defined for analysis (dotted white line). Representative images of cells seeded on PLL-PEG-biotin + Strep-αIgM Fab coated glass slides and activated for 30 minutes stained for F-actin (white outline) and the corresponding phospho-protein (magenta) are shown. Scale bar 5µm. Ratio of the periphery RFI/ Center (or pattern) RFI of phospho-proteins in all time points and conditions. Violin plot graphs with mean (central line), quartiles (top and bottom lines) and experiment means (dots) are shown. One-way ANOVA of experiment means (N=3 experiments, n=50-103 cells) (*P < 0.05, **P < 0.01, ***P < 0.001, ****P < 0.0001).

Journal of Cell Science • Supplementary information

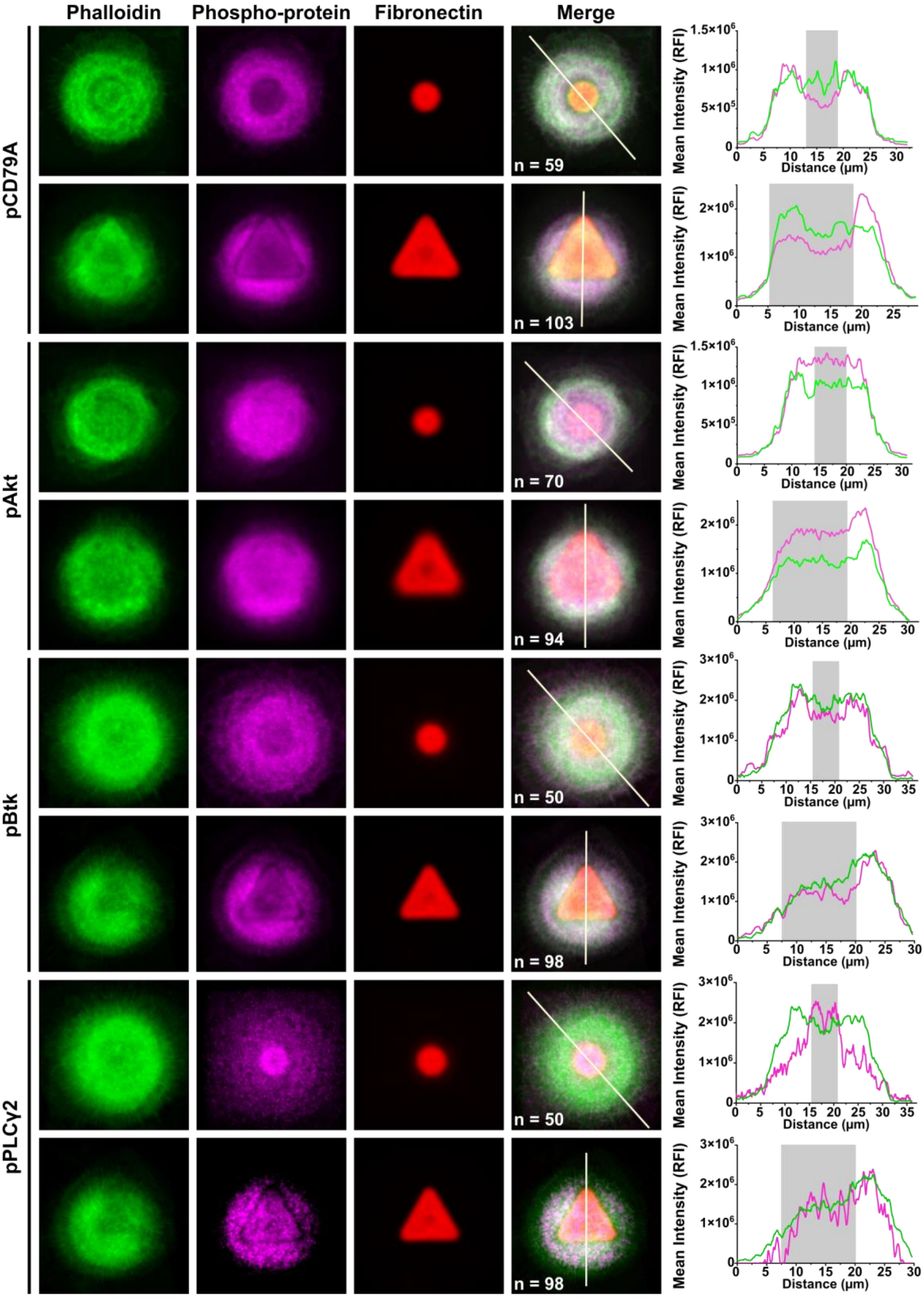

Fig. S3. Analysis of different localization patterns on dynamic micropatterns. All images of single confocal microscopy slices of B cells activated with Strep-αIgM Fab for 30 minutes on 5 µm circular or 15 µm triangle dynamic micropatterns, fixed and stained for F-actin (green) and phospho-proteins (magenta), were aligned using the fibronectin/fibrinogen-AF546 signal. The phospho-protein summed images are also shown in Figs 2A-C and 3A-C for the 30 minute timepoints. The pBtk and pPLCγ2 images were created using the same image set. Summed images of all cells per condition were created using Fiji. Histograms represent mean intensity of F-actin (green) and phospho-proteins (magenta) along the white line drawn on merge images, grey background area marks the section that transverses the micropattern.

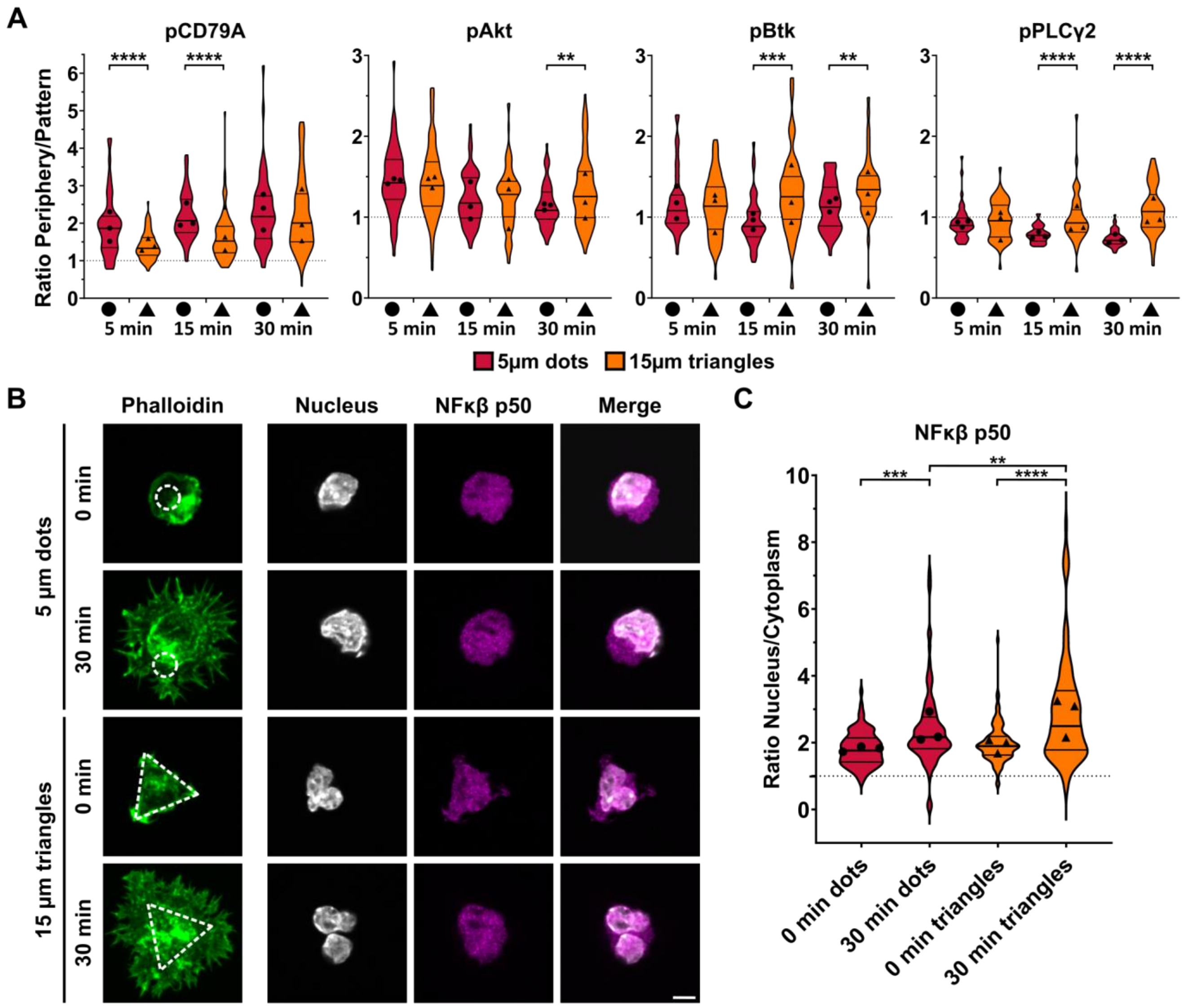

Fig. S4. Signaling protein localization and NFkB nuclear translocation in circular and triangular micropatterns. (A) The ratio of periphery RFI/ pattern RFIpCD79ApAktpBtk and pPLCγ2 comparing the 15 µm triangle micropatterns (Figure 3 data A-C) to the 5 µm circular (Figure 2 data A-C). Both sets of experiments were performed at different times. Violin plot graphs with mean (central line), quartiles (top and bottom lines) and experiment means (dots) are shown. Unpaired t-test (N=3 experiments, n=48-131 cells). (B) A20 D1.3 B cells were allowed to form immune synapses via first seeding them on fibronectin/fibrinogen-coated circular 5 µm or triangular 15 µm (dotted white line) dynamic micropatterns for 60 minutes and then activated by the addition of Strep-αIgM Fab for 0 or 30 minutes. Samples were fixed and stained for immunofluorescence analysis to visualize F-actin (phalloidin, green), nucleus (white) and NFKB (magenta). Representative images of full stack projections (phalloidin) or of single planes of the stack (nucleus and NFKB) are shown. Scale bar 5 µm. (C) The ratio of Nucleus RFI/ Cytoplasm RFI of a slice in the center of the nucleus in all time points and micropattern shapes from the data in B. Violin plot graphs with mean (central line), quartiles (top and bottom lines) and experiment means (dots) are shown. One-way ANOVA (N=3 experiments, n=97- 110 cells) (*P < 0.05, **P < 0.01, ***P < 0.001, ****P < 0.0001).

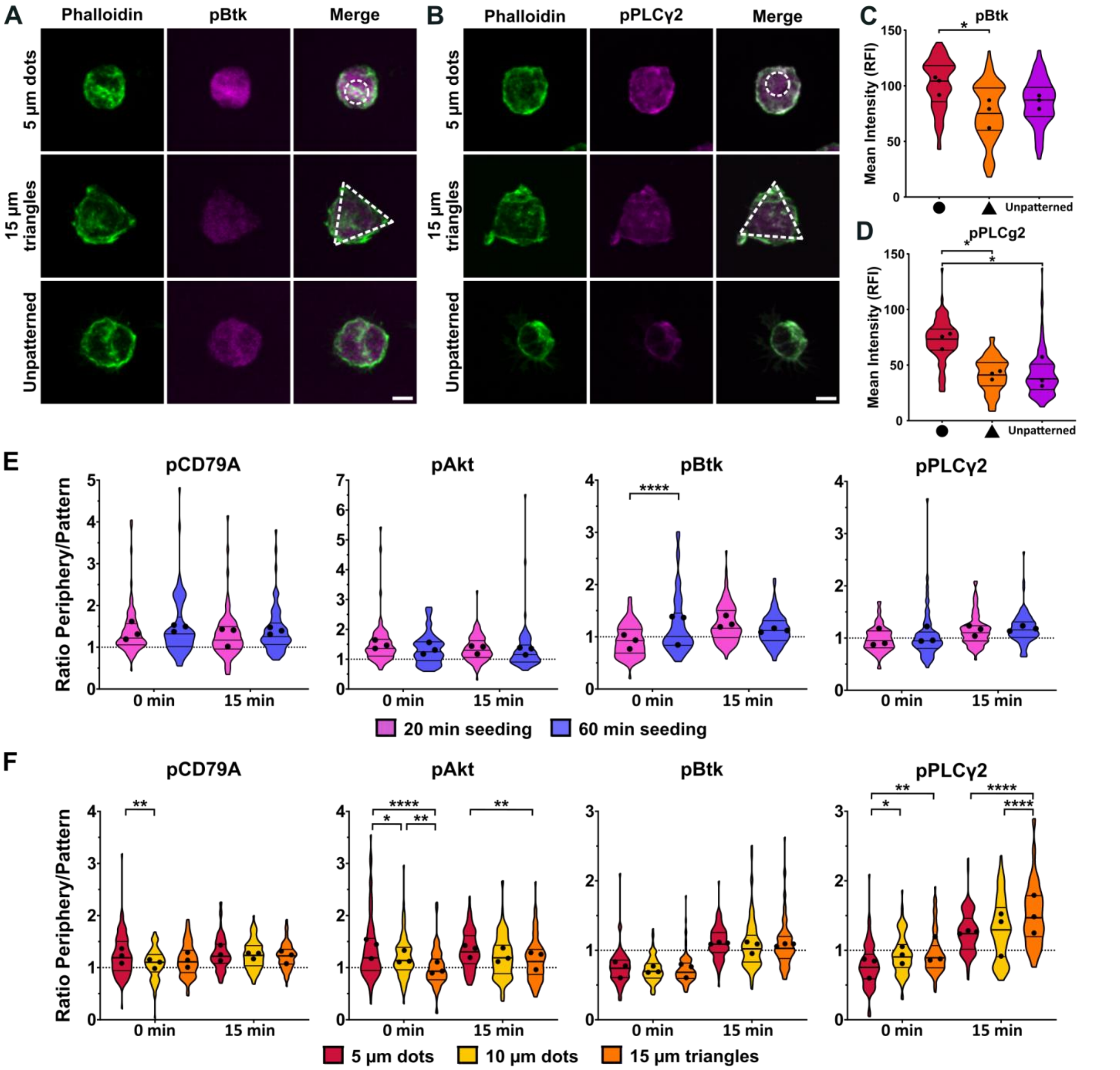

Fig. S5. Different seeding platforms affect BCR signalling. (A, B) A20 D1.3 B cells were seeded on fibronectin/ fibrinogen-coated circular 5 µm or triangular 15 µm (dotted white line) dynamic micropatterns or fibronectin/ fibrinogen-coated glass for 1 hour and then fixed and stained for immunofluorescence analysis to visualize F-actin (green) and pBtk (magenta, A) or pPLCγ2 (magenta, B). Representative images of single confocal microscopy slices are shown. Scale bar 5 µm. (C, D) Mean intensities of pBtk and pPLCγ2 in all seeding platforms from the data in A,B. Violin plot graphs with mean (central line), quartiles (top and bottom lines) and experiment means (dots) are shown. One-way ANOVA of experiment means (N=3 experiments, n=93-156 cells). (E) A20 D1.3 B cells were let to form immune synapses via seeding them on fibronectin/fibrinogen-coated circular 5 µm dynamic micropatterns for 20 minutes and activated or left in the micropatterns for 40 minutes more and then activated by the addition of Strep-α IgM Fab for 0 or 15 minutes. Samples were fixed and stained for immunofluorescence analysis to visualize F-actin and pCD79A, pAkt, pBtk and pPLCγ2. The ratio of periphery RFI/ pattern RFI of pCD79ApAktpBtk and pPLCγ2 comparing short and long pre-adhesion times. Violin plot graphs with mean (central line), quartiles (top and bottom lines) and experiment means (dots) are shown. One-way ANOVA (N=3 experiments, n= 76-100 cells). (F) A20 D1.3 B cells were let to form immune synapses via seeding them on fibronectin/fibrinogen-coated circular 5 µm, circular 10 µm or 15 µm triangular dynamic micropatterns for 60 minutes and activated by the addition of Strep-αIgM Fab for 0 or 15 minutes. Samples were fixed and stained for immunofluorescence analysis to visualize F-actin and pCD79ApAkt, pBtk and pPLCγ2. The ratio of periphery RFI/ pattern RFI of pCD79ApAktpBtk and pPLCγ2 comparing different pattern shapes. Violin plot graphs with mean (central line), quartiles (top and bottom lines) and experiment means (dots) are shown. One-way ANOVA (N=3 experiments, n=92-138 cells) (*P < 0.05, **P < 0.01, ***P < 0.001, ****P < 0.0001).

Journal of Cell Science • Supplementary information

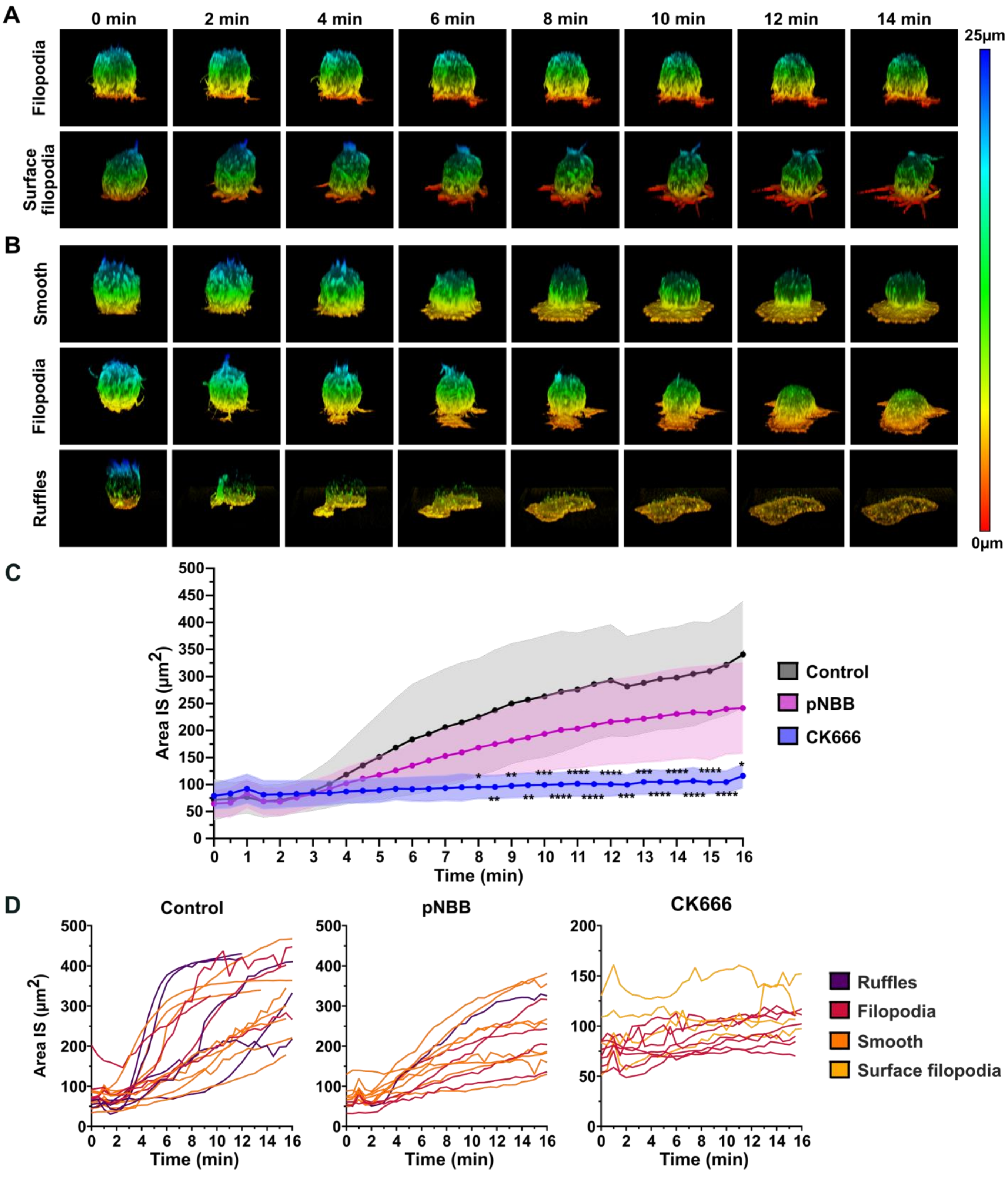

Fig. S6. Spreading phenotypes and spreading area vary in B cells treated with cytoskeleton inhibitors. (A-B) A20 D1.3 B cells, transfected with LifeAct-Emerald, were seeded on fibronectin/fibrinogen-coated circular 5 µm dynamic micropatterns for 20 minutes, treated with the inhibitors CK666 (A) or pNBB (B), and activated for 15 minutes by the addition of Strep-αIgM Fab with simultaneous imaging with Leica Stellaris confocal microscope. Representative images of 3D confocal live imaging show different phenotypes: smooth spreading, filopodia-driven spreading, surface filopodia-driven spreading and actin ruffles-driven spreading. (C-D) The IS of B cells imaged live (Figure 4) were analysed for their spreading area. (C) Line graph with standard deviation of the mean area of the IS in each time point since activation of control (grey), pNBB treated (pink) and CK666 treated (blue) cells. One-way ANOVA (n=11-17 cells per condition) (*$P < 0.05$, **$P < 0.01$, ***$P < 0.001$, ****$P < 0.0001$). (D) Line graph of the area of the IS in each time point after activation. Control cells not treated with the inhibitors (left), and pNBB (center) and CK666 (right) treatments, colour coded for the different spreading phenotypes: ruffle (purple), filopodia (red), smooth spreading (orange) and surface filopodia-driven spreading (yellow).

Journal of Cell Science • Supplementary information

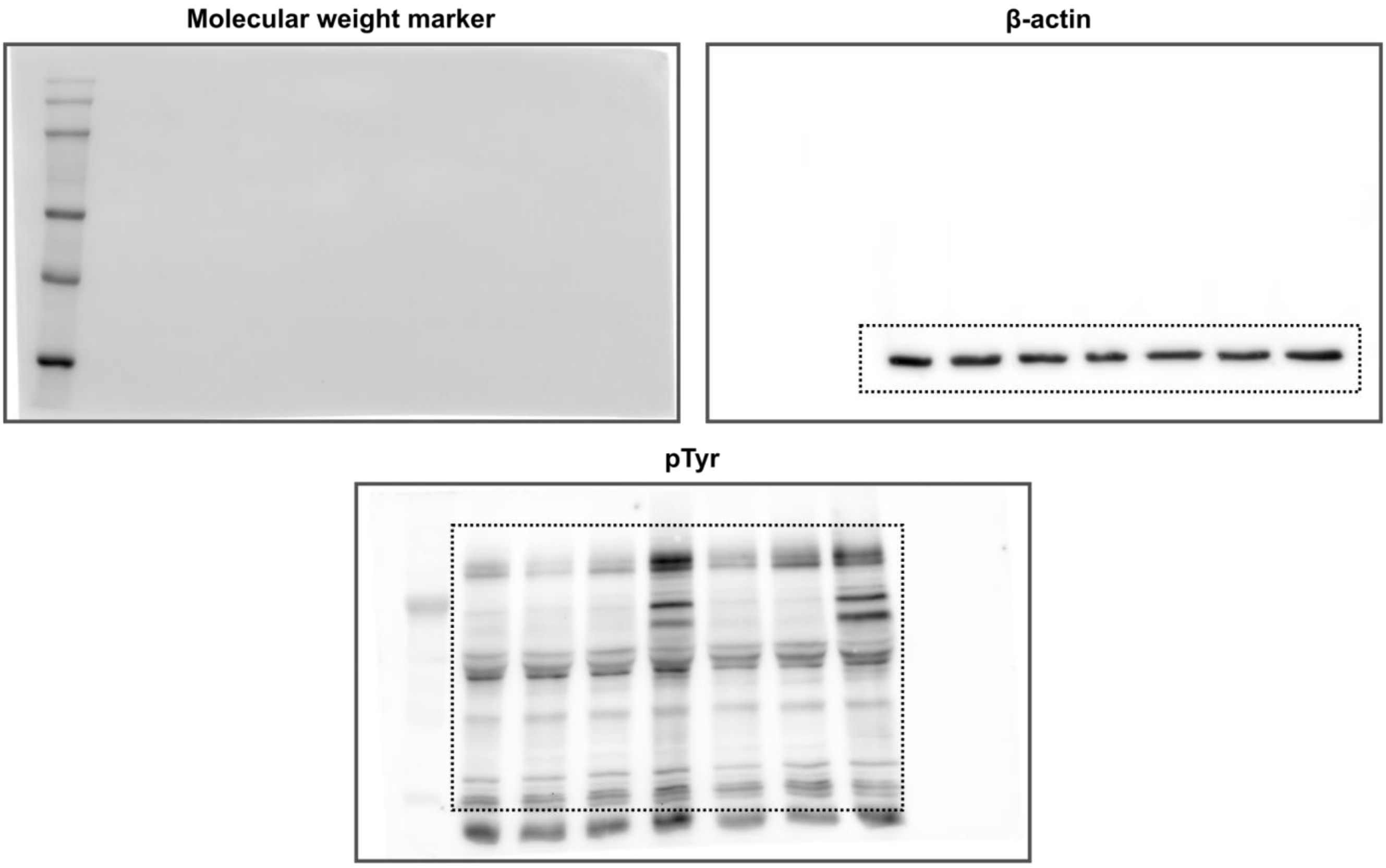

Fig. S7. Blot transparency. Images corresponding to uncropped immunoblot in Supplementary Figure S1. Marked areas show the cropping used in the figure.

Journal of Cell Science • Supplementary information

Table S1. List of antibodies used for immunofluorescence, western blotting and B cell activation.

Antibody	Commercial house	Catalog number	Source species	Clone	Dilution
AF488 pPLCg2 (Y759)	BD Biosciences	558507	Mouse	K86-689.37	1:100
AF647 pBtk (y223)/pIt(Y180)	BD Biosciences	564846	Mouse	N35-86	1:100
Anti-phosphotyrosine	Merck	05-321	Mouse	4G10	1:1000
Donkey anti-Goat IgG (H+L) Cross-Adsorbed Secondary Antibody, Alexa Fluor 488	Thermo Fisher Scientific	A-11055	Donkey	Polyclonal	1:500
Donkey anti-Rabbit IgG (H+L) AF647	Invitrogen	A31573	Donkey	Polyclonal	1:500
$F(ab')_2$ Fragment Goat Anti-Mouse IgM, μ chain specific	Jackson ImmunoResearch	115-006-020	Goat	Polyclonal	
Fab Fragment Goat Anti-Mouse IgM, μ Chain Specific	Jackson ImmunoResearch	115-007-020	Goat	Polyclonal	
HRP Anti-beta actin antibody	Abcam	#ab49900	Mouse	AC-15	1:20000
pAKT (Ser473)	Cell Signal Technology	4058	Rabbit	193H12	1:100
pCD79A (Tyr182)	Cell Signal Technology	5173	Rabbit	Polyclonal	1:100
NFκB p50 AF488	Santa Cruz Biotechnology	sc-8414	Mouse	Monoclonal	1:100

Journal of Cell Science • Supplementary information

Table S2. List of reagents and consumables used for experimental work.

Product	Commercial house	Catalog number
13 mm diameter coverslips	Epredia	CB00130RAC20MNZ0
25 mm diameter coverslips	Epredia	CB00250RAC20MNZ0
ActiStain 488 Phalloidin	Cytoskeleton	PHDG1-A
ActiStain 555 Phalloidin	Cytoskeleton	PHDH1-A
Attofluor cell chamber for microscopy	Invitrogen	A7816
CK666	Sigma-Aldritch	182515
Fibronectin	Sigma Aldritch	F4759
Fibronectin bovine plasma	Sigma-Aldritch	F4759
Fluoromount G	Southern Biotech	0100-01
Fluoromount G with DAPI	Invitrogen	00495952
Immobilon Western Chemiluminescent HRP Substrate	Merck	WBKLS0500
Latrunculin A	Merck Millipore	428021
Molecular Probes Fibrinogen From Human Plasma, Alexa Fluor 546 Conjugate	Thermo Fisher Scientific	F13192
Paraformaldehyde	Sigma-Aldritch	16005
Para-nitro-Blebbistatin	Cayman Chemical	24171
Phalloidin AF488	Cell Signalling Technology	8878S
Phalloidin-iFluor 405 reagent	Abcam	ab176752
PLL(20)-g[3.5]- PEG(2)/PEG(3.4)- biotin(50%)	SuSoS	
Recombinant mouse ICAM-1-Fc Chimera (carrier-free)	BioLegend	553004
Streptavidin conjugation kit	Abcam	ab102921
12 well 5mm numbered slides	Epredia	X1XER302W

Journal of Cell Science • Supplementary information

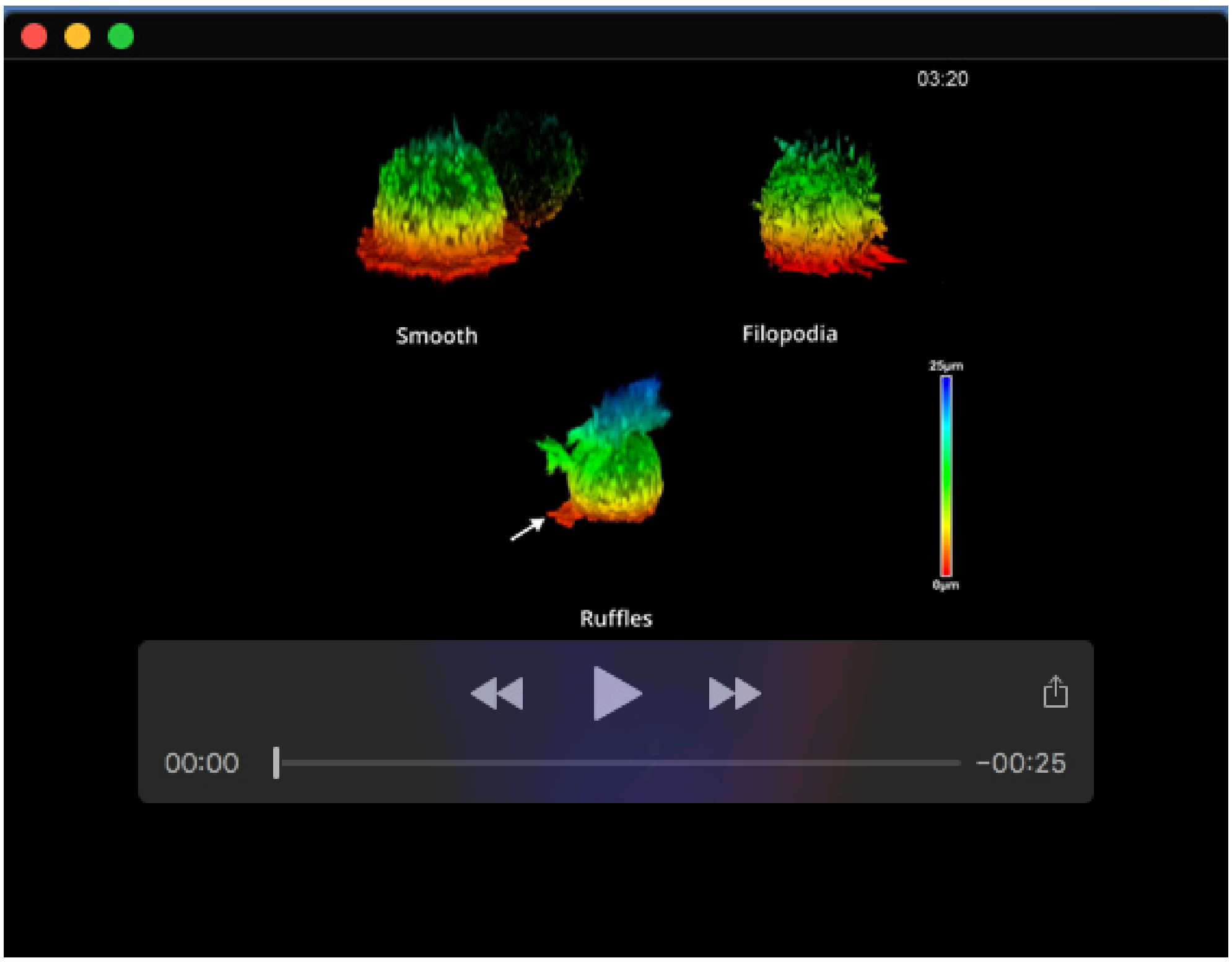

Movie 1. Different spreading phenotypes observed upon B cell IS formation. Videos of different spreading phenotypes corresponding to Figure 4A. The cells were imaged with Leica Stellaris confocal microscope every 30 seconds. Representative videos of 3D confocal live imaging show different phenotypes: smooth spreading (top left), filopodia-driven spreading (top right), and actin ruffle-driven spreading (bottom). Scale bar 25µm.

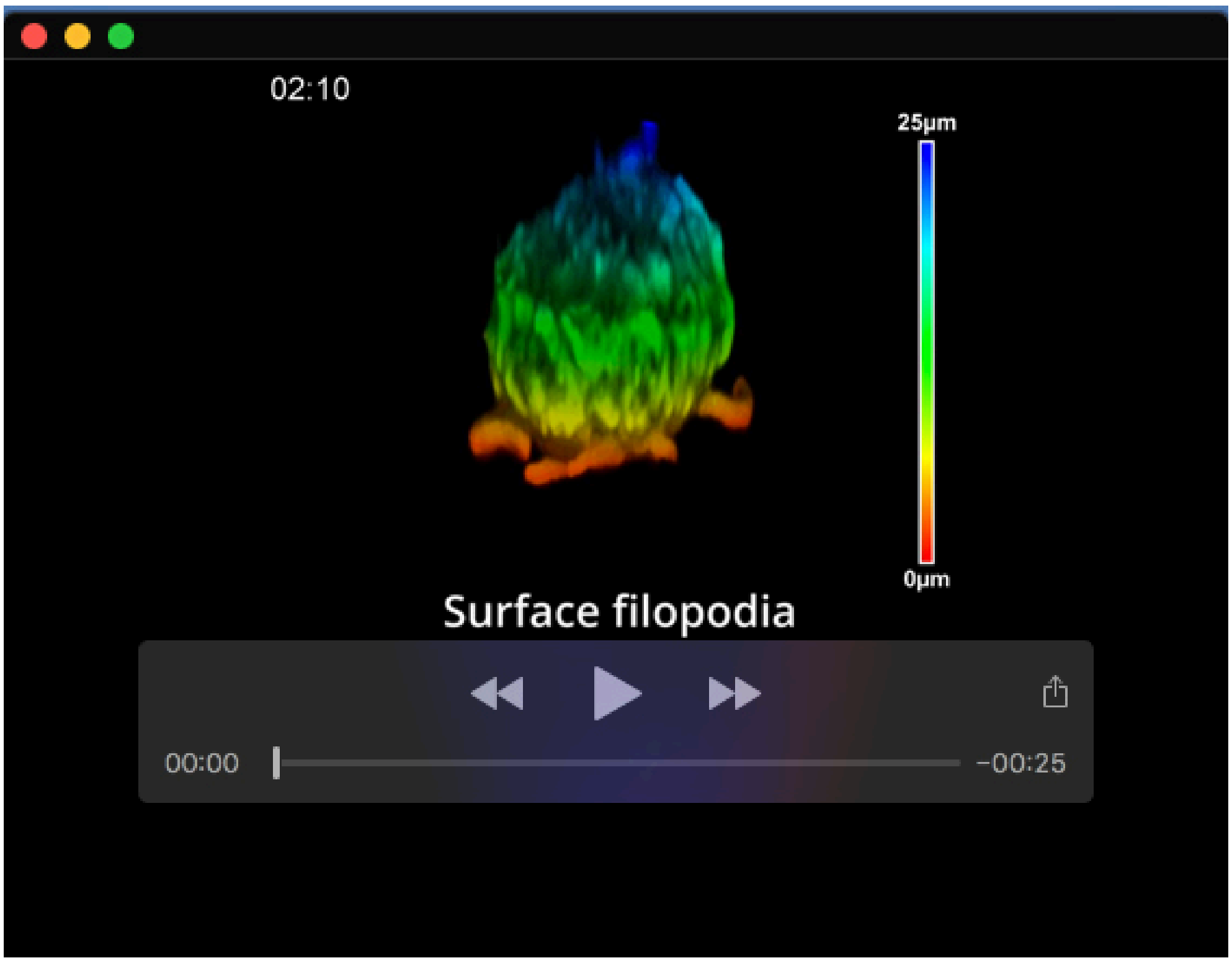

Movie 2. Surface filopodia phenotype in Arp2/3 inhibited cells. Representative video of 3D confocal live imaging, corresponding to Figure 4C, showing the surface filopodia-driven spreading upon treatment with CK666. The cells were imaged with Leica Stellaris confocal microscope every 30 seconds. Scale bar 25µm.

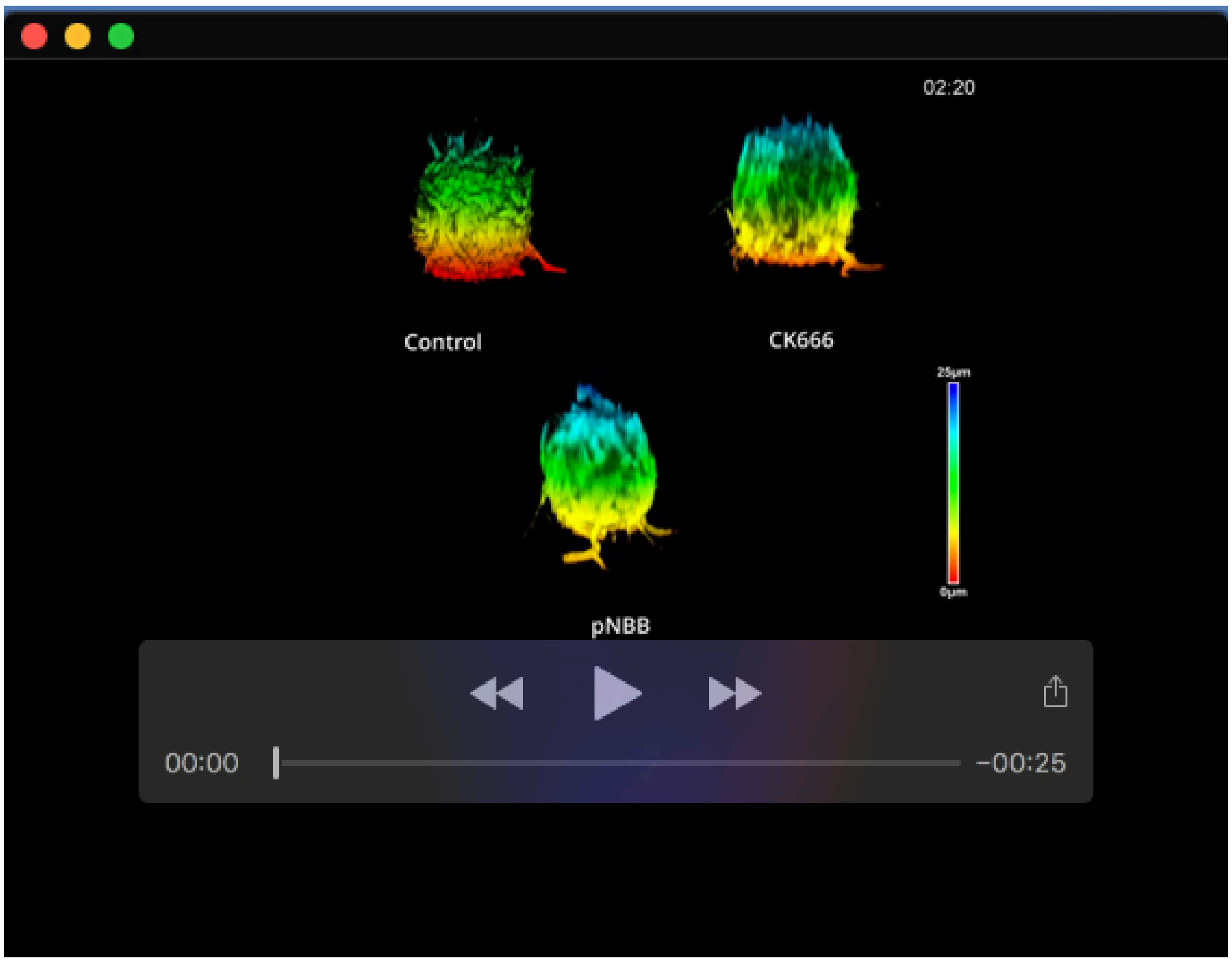

Movie 3. Comparison of filopodia phenotypes in cells treated with cytoskeletal inhibitors. Representative videos of 3D confocal live imaging, corresponding to Figure 4D, show filopodia-driven spreading in control (top left), CK666 treated (top right), and para-nitro-blebbistatin treated cells (bottom). The cells were imaged with Leica Stellaris confocal microscope every 30 seconds. Scale bar 25µm.

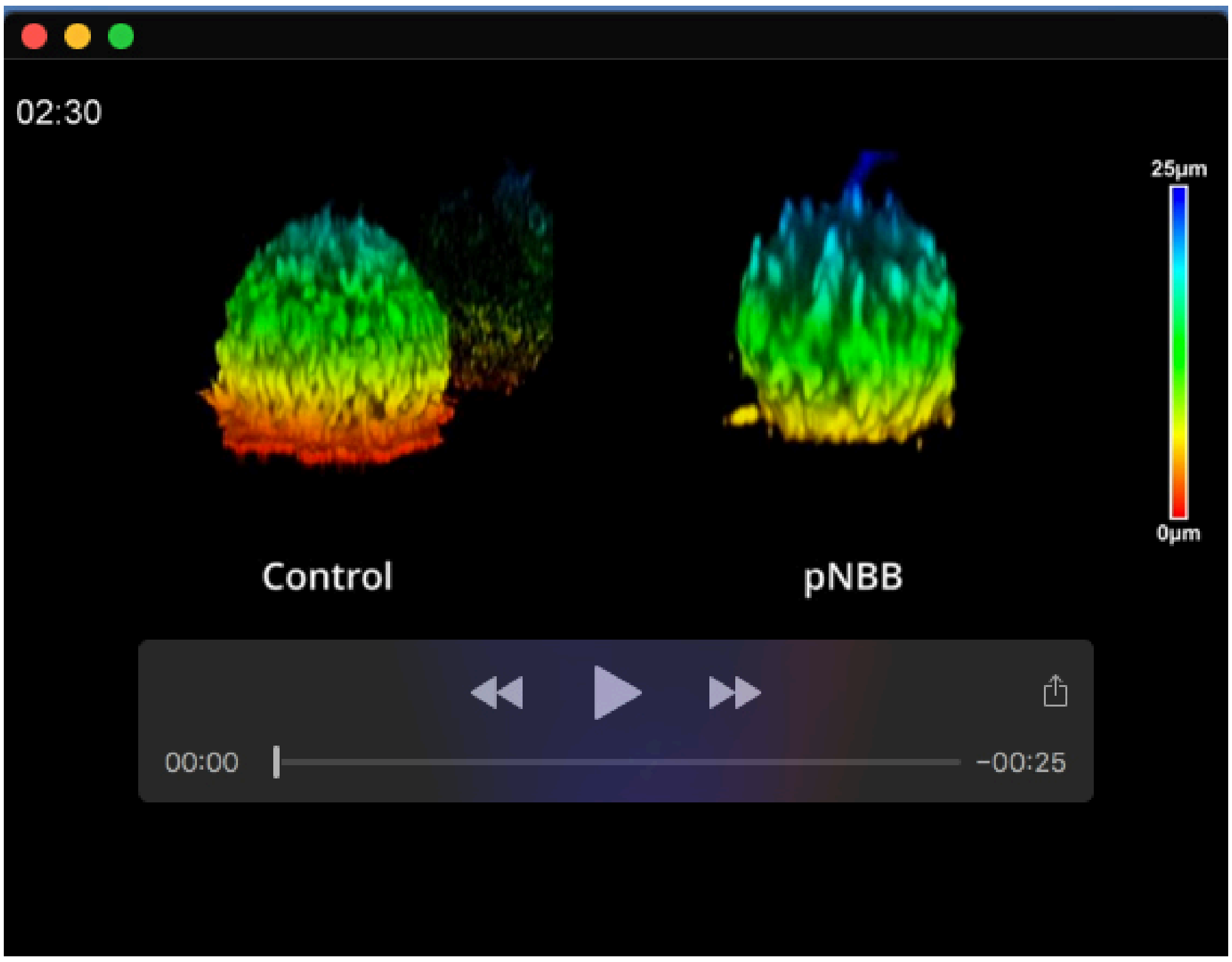

Movie 4. Comparison of smooth phenotype in cells treated with cytoskeletal inhibitors. Representative videos of 3D confocal live imaging, corresponding to Figure 4D, show smooth spreading in control, and para-nitro-blebbistatin treated cells (left to right). The cells were imaged with Leica Stellaris confocal microscope every 30 seconds. Scale bar 25µm.

Journal of Cell Science • Supplementary information

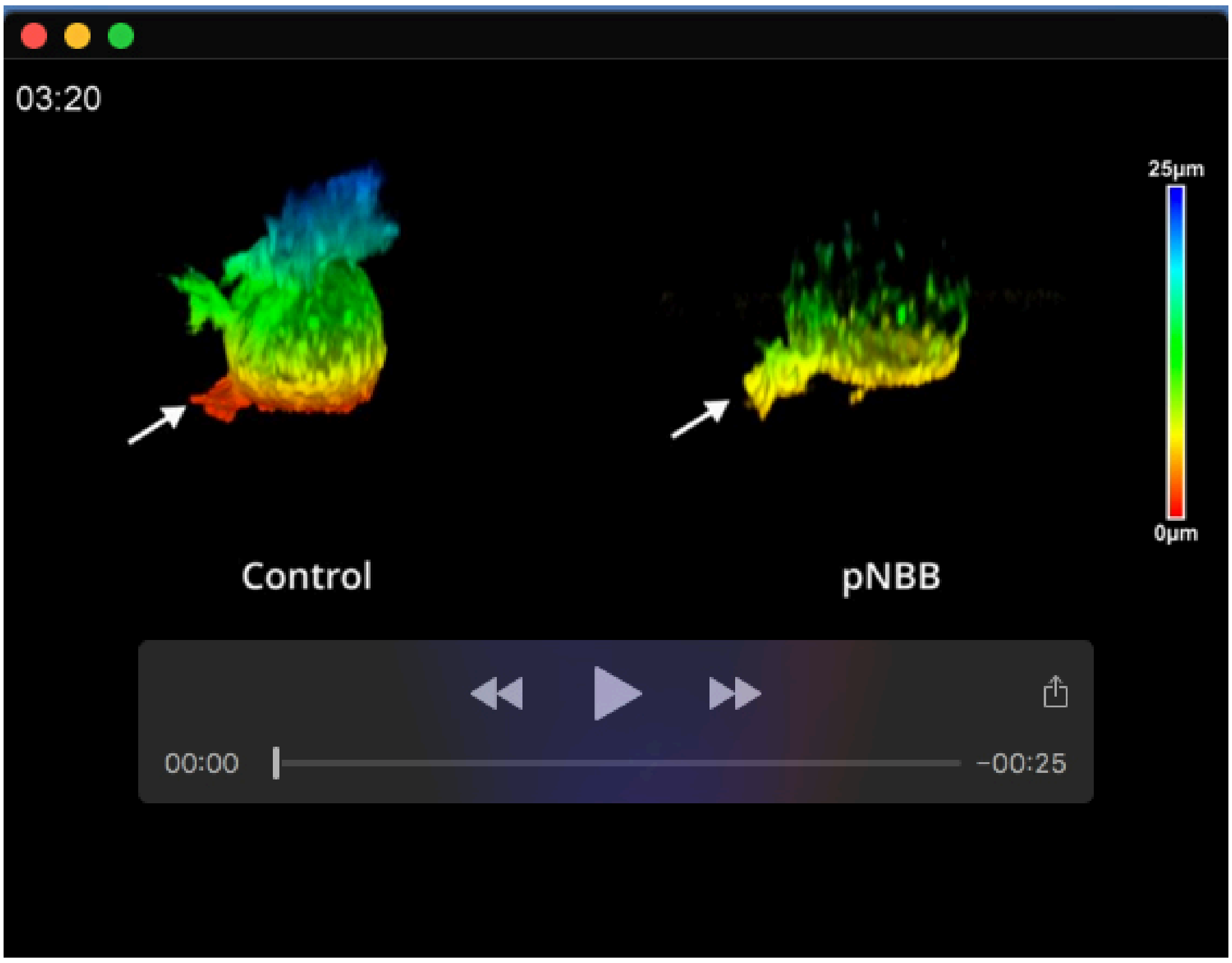

Movie 5. Comparison of ruffle phenotype in cells treated with cytoskeletal inhibitors. Representative videos of 3D confocal live imaging, corresponding to Figure 4D, show actin ruffle-driven spreading in control, and para-nitro-blebbistatin treated cells (left to right). The cells were imaged with Leica Stellaris confocal microscope every 30 seconds. Scale bar 25µm.

Journal of Cell Science • Supplementary information
